# Exercise prescription for axial spondyloarthritis: a systematic review and meta-analysis of randomized controlled trials

**DOI:** 10.3389/fmed.2026.1783569

**Published:** 2026-02-20

**Authors:** Chunhui Yu, Lina Guo, Xiaoxiao Gao, Xiaojie Shen, Xilan Ma, Ji Li, Weifei Wu, Junhao Cai

**Affiliations:** 1 The First Clinical College, Liaoning University of Traditional Chinese Medicine, Shenyang, China; 2 Department of Rehabilitation Medicine, Caoxian People’s Hospital, Heze, China; 3 Shanghai Jiading District Zhenxin Community Health Service Center, Shanghai, China; 4 Jiading Industrial Zone Community Health Service Center, Shanghai, China; 5 The Third Department of Orthopedics and Traumatology, Liaoning Second Hospital of Traditional Chinese Medicine Affiliated to Liaoning University of Traditional Chinese Medicine, Shenyang, China; 6 School of Computer Science and Engineering, Northeastern University, Shenyang, China; 7 Shanghai Municipal Hospital of Traditional Chinese Medicine, Shanghai University of Traditional Chinese Medicine, Shanghai, China

**Keywords:** ankylosing spondylitis, exercise therapy, meta-analyses, prescription, randomized controlled trials, systematic reviews

## Abstract

**Background:**

Axial spondyloarthritis (axSpA) is a chronic inflammatory rheumatic condition that significantly impacts patients’ quality of life. Exercise therapy serves as a core non-pharmacological treatment modality, yet its overall efficacy and optimal prescription parameters require further clarification through high-quality evidence. This study aims to systematically evaluate the efficacy of exercise interventions for axSpA patients.

**Methods:**

Computerized searches were conducted across databases including PubMed, Embase, Web of Science, and the Cochrane Library, covering the period from inception to September 2025. Randomized controlled trials (RCTs) comparing exercise interventions with conventional treatments for axSpA were included. Two researchers independently performed literature screening, data extraction, and quality assessment. The heterogeneity of the research results was assessed using the *I*^2^ statistic. Continuous variables were presented as weighted mean differences or standard mean differences, with confidence intervals set at 95%. Stata 15.0 was utilized to conduct a meta-analysis.

**Results:**

Fifteen RCTs involving 1,699 patients were included. Meta-analysis revealed that exercise intervention significantly improved disease activity in axSpA patients compared with controls (BASDAI: SMD = –0.75, 95% CI: –1.19 to –0.31; ASDAS: SMD = –0.91, 95% CI: –1.54 to –0.29), physical function (BASFI: SMD = –0.37, 95% CI: –0.47 to –0.26), spinal mobility (BASMI: SMD = –0.26, 95% CI: –0.49 to –0.04), thoracic expansion (SMD = 0.35, 95% CI: 0.04–0.65), and fatigue levels (SMD = –0.53, 95% CI: –0.78 to –0.28). Subgroup analyses indicated that different exercise modalities and intervention durations influenced treatment efficacy.

**Conclusion:**

This meta-analysis confirms that exercise interventions significantly improve core outcome measures including disease activity, physical function, spinal mobility, and fatigue in patients with axial spondyloarthritis, with statistically significant effects. The findings support the incorporation of individualized exercise prescriptions as a key component of standard axSpA treatment, providing evidence-based guidance for clinical practice. Future research should further optimize exercise prescription parameters and validate their long-term efficacy.

**Systematic review registration:**

https://www.crd.york.ac.uk/PROSPERO/view/CRD420251144518, identifier CRD420251144518.

## 1 Introduction

Axial spondyloarthritis (axSpA) is a group of chronic inflammatory rheumatic diseases primarily affecting the spine and sacroiliac joints, encompassing ankylosing spondylitis (AS) and non-radiographic axial spondyloarthritis (nr-axSpA). Its core clinical feature is inflammatory low back pain. This typically presents before the age of 45, improves with exercise but does not resolve with rest, and may be accompanied by alternating hip pain (1). Furthermore, morning stiffness and fatigue are common symptoms in axSpA patients, significantly impacting quality of life and daily functioning (2, 3). Some individuals may also present with extra-articular manifestations such as peripheral arthritis, enthesitis and uveitis, inflammatory bowel disease, or psoriasis, further increasing disease burden (4–6). As the etiology of axSpA remains incompletely understood and no curative treatment exists, patients often require lifelong management. This not only impacts quality of life but also imposes a substantial burden on society and healthcare systems. Epidemiological data indicate a global prevalence of axSpA ranging from 0.3 to 1.4% (7), with marked geographical variation (8). Male prevalence consistently exceeds that of females, with onset typically occurring during young adulthood, leading to long-term limitations in working capacity and diminished quality of life (8). Furthermore, data from the Greek AxSpA Registry Study, involving 717 patients, revealed a male predominance of 64.9% (9). This finding further substantiates the male-dominated prevalence pattern in axSpA.

Current treatment strategies primarily encompass two categories: pharmacological and non-pharmacological approaches. Non-steroidal anti-inflammatory drugs (NSAIDs) represent the first-line therapy, effectively alleviating pain and stiffness (10, 11). Should NSAIDs prove insufficient, biologics such as TNF-α inhibitors and IL-17 inhibitors become crucial alternatives, having demonstrated significant improvement in disease activity and radiographic progression (12–15). However, some patients exhibit poor drug response, and long-term medication may carry adverse effects including cardiovascular, infectious, and metabolic complications (12). Consequently, non-pharmacological therapies, particularly exercise therapy, have gained increasing prominence in recent years. As a core non-pharmacological intervention in axSpA management, exercise intervention offers advantages of high safety, minimal side effects, and strong feasibility (16). Exercise, as a central non-pharmacological intervention in axSpA management, exerts its effects through multiple synergistic pathways. These include modulating immune-inflammatory responses, improving musculoskeletal function, enhancing cardiopulmonary fitness, and alleviating fatigue. The combined action of these mechanisms establishes exercise as a vital component of comprehensive axSpA management (17–19).

According to the World Health Organization (WHO) 2020 Guidelines on Physical Activity and Sedentary Behavior, physical activity should encompass two core forms: ➀ Structured exercise (such as planned activities including aerobic training, strength training, and yoga); ➁ Physical activity during daily routines (such as unplanned activities including walking to work, climbing stairs, domestic chores, and gardening) (20). Despite existing evidence indicating significant benefits of exercise interventions for axial spondyloarthritis (axSpA), several limitations remain: most studies feature limited sample sizes and short follow-up periods, lacking support from multicenter, large-scale randomized controlled trials (RCTs); optimal prescription parameters for exercise type, intensity, and duration remain unclear; moreover, previous meta-analyses predominantly focused on disease activity and function, with insufficient systematic assessment of key outcomes such as thoracic expansion and fatigue. Therefore, this study employs a systematic review and meta-analysis to synthesize recent multinational RCT evidence, comprehensively evaluating the multidimensional efficacy of exercise therapy for axSpA patients. It further explores the differential impact of exercise modality and prescription parameters on clinical outcomes, providing evidence-based support for the development of individualized exercise prescriptions.

## 2 Methods

This study has been registered with the PROSPERO International Prospective Systematic Reviews Register (CRD420251144518). It was conducted in accordance with the Cochrane Handbook for Systematic Reviews and followed the Preferred Reporting Items for Systematic Reviews and Meta-Analyses (PRISMA) statement (21).

### 2.1 Inclusion criteria

The Population-Intervention-Control-Outcome-Study design (PICOS) framework was employed as the inclusion criteria for this evaluation, as detailed below.

#### 2.1.1 Selection of studies

All randomized controlled trials (RCTs) investigating exercise therapy for axial spondyloarthritis (axSpA), without language restrictions.

#### 2.1.2 Selection of participants

The study population comprised patients clinically diagnosed with active axial spondyloarthritis (axSpA). All participants met the international classification criteria and had a BASDAI score ≥ 3.5, with no restrictions on subtype (radiographic/non-radiographic) or type of therapeutic medication.

#### 2.1.3 Types of interventions

All participants included in the study were patients with axial spondyloarthritis (axSpA), encompassing both ankylosing spondylitis (AS) and non-radiographic axial spondyloarthritis (nr-axSpA). The intervention group received exercise therapy interventions, with exercise forms including but not limited to cardiopulmonary training and strength training. No restrictions were imposed on exercise selection, permitting either single or combined modalities. The control group received standard care, health education, or maintained their usual lifestyle. Standard care may encompass medication maintenance, outpatient follow-up, or rehabilitation support without exercise intervention. Studies failing to meet the above intervention definition were excluded. Additional exclusion criteria were: ➀ non-randomized controlled trials; ➁ interventions not primarily exercise-based, or combined with other physical therapies, electrical stimulation, or enhanced pharmacological treatments, where the independent effect of exercise therapy could not be extracted; ➂ participants with other major complications (e.g., severe cardiopulmonary disease, neurological disorders) affecting intervention outcomes; ➃ studies lacking raw data or presenting incomplete research data. Supervision methods and venues for exercise interventions are categorized into three types: ➀ Hospital-supervised exercise: On-site guidance by professional rehabilitation specialists/therapists, conducted in hospital rehabilitation departments, sports centers, or designated medical facilities, with continuous or periodic supervision throughout the intervention; ➁ Digitally supervised exercise (digital intervention): Remote supervision without on-site guidance, achieved through digital means such as video consultations, app check-ins, and online course delivery, with exercise taking place at the patient’s home or community; ➂Home-based independent exercise: No professional supervision; patients complete exercises autonomously following written/verbal protocols. Exercise takes place at home.

### 2.2 Outcome measures

By reviewing randomized controlled trials published in major databases and academic journals, this paper summarizes and distills commonly used outcome measures for evaluating exercise therapy interventions in patients with axial spondyloarthritis (axSpA). Analysis reveals that primary outcome measures focus on disease activity scores, chiefly comprising the Bath Ankylosing Spondylitis Disease Activity Index (BASDAI) and the Ankylosing Spondylitis Disease Activity Score (ASDAS). These metrics reflect changes in comprehensive symptoms including inflammatory activity levels, pain intensity, and fatigue. Secondary outcome measures encompass the following domains: Physical function assessment: The Bath Ankylosing Spondylitis Functional Index (BASFI) is commonly employed to gauge functional limitations in daily living activities. Spinal mobility assessment: The Bath Ankylosing Spondylitis Metrology Index (BASMI) objectively measures range of motion in the spine and hips. Thoracic expansion: Assessing thoracic stiffness by measuring respiratory amplitude, serving as a key indicator of respiratory function limitation; Fatigue severity: Some studies employ the Visual Analog Scale for Fatigue (VAS Fatigue) or the Fatigue Severity Scale (FSS) to evaluate improvements in chronic fatigue symptoms following intervention. These outcome measures are extensively employed in exercise therapy intervention studies, comprehensively reflecting changes in axSpA patients regarding inflammation control, functional improvement, physical flexibility, and subjective wellbeing.

### 2.3 Database search

Computerized searches were conducted in PubMed, Embase, Web of Science, and Cochrane, covering the period from indexing to September 2025. The search strategy employed the following keywords: “axial spondyloarthritis (axSpA), ankylosing spondylitis, exercise therapy, physical activity, exercise intervention.” Using PubMed as an example, the search terms and strategy were as follows: [(“Spondylarthritis”[MeSH Terms] OR (“Spondylarthritis”[MeSH Terms] OR “Spondylarthritis”[All Fields] OR “spondylarthritides”[All Fields]) OR “arthritis spinal”[All Fields] OR “Spinal Arthritis” [All Fields] OR “Spinal Arthritides”[All Fields]) AND (“Exercise”[MeSH Terms] OR “Exercise Therapy”[MeSH Terms] OR “Physical Exertion”[MeSH Terms] OR (“Exercise”[MeSH Terms] OR “Exercise” [All Fields] OR “exercises”[All Fields] OR “Exercise Therapy”[MeSH Terms] OR (“Exercise” [All Fields] AND “therapy”[All Fields]) OR “Exercise Therapy”[All Fields] OR “exercising”[All Fields] OR “exercise s”[All Fields] OR “exercised”[All Fields] OR “exerciser”[All Fields] OR “exercisers”[All Fields]) OR “Physical Exercise” [All Fields] OR “Aerobic Exercise”[All Fields] OR “Isometric Exercise”[All Fields] OR “Acute Exercise”[All Fields] OR “Exercise Training”[All Fields] OR “Physical Activity”[All Fields])] AND [randomisedcontrolledtrial (Filter)].

### 2.4 Literature screening and data extraction

Two researchers (CHY, WFW) independently screened the literature according to predefined inclusion and exclusion criteria to ensure rigor and consistency in the screening process. All retrieved literature was managed and deduplicated using ENDNOTE software. Subsequently, an Excel spreadsheet was utilized to create a data extraction table for the 15 included studies, facilitating data extraction and collation. Key extracted elements included: study design, sample size, mean age of participants, disease type and diagnostic criteria, disease duration, intervention method and duration, control group measures, primary and secondary outcome measures (e.g., BASDAI, ASDAS, BASFI, BASMI, chest expansion, fatigue scores). Finally, results underwent cross-checking, with disputes resolved through discussion or third-party consultation. Where raw data were unavailable or presented graphically, attempts were made to contact relevant authors for raw data, supplemented by data extraction from graphs using GetData Graph Digitizer 2.26 software.

### 2.5 Quality assessment

The quality of included studies was assessed using the Cochrane Handbook for Systematic Reviews, Chapter 5.3, Offset Risk Assessment Tool, whilst RevMan software was employed to evaluate the methodological quality of articles. Assessment components comprised: (1) random sequence generation; (2) allocation concealment; (3) blinding of participants and personnel; (4) blinding of outcome assessors; (5) completeness of outcome data; (6) selective reporting of results; (7) other biases. Assessment results were categorized as “low risk,” “risk of bias unclear,” or “high risk.”

### 2.6 Certainty assessment

GDT software (22) was used to assess the certainty of evidence according to the GRADE guidelines (gradeworkinggroup.org) for primary outcomes based on areas of study design, risk of bias, inconsistency, indirectness, imprecision, and other considerations, such as publication bias, effect size, and potential confounding. And the quality of the final evidence was classified as high, moderate, low, and very low (23, 24).

### 2.7 Statistical analysis

Meta-analyses were conducted using Stata 15.0 software. Continuous variables were analyzed using the inverse variance method, with weighted mean difference (WMD) as the effect measure. When absolute differences in continuous variables were substantial or units were inconsistent, standardized mean difference (SMD) was employed as the effect measure. Confidence intervals (CI) were set at 95%. The *I*^2^ statistic was employed to assess heterogeneity among studies. Where *I*^2^ ≤ 50%, results from fixed-effects (FE) models were analyzed; where *I*^2^ > 50%, results from random-effects (RE) models were pooled. Subgroup analyses explored sources of heterogeneity, whilst sensitivity analyses evaluated the robustness of meta-analysis outcomes. Publication bias was examined using funnel plots.

## 3 Results

### 3.1 Research findings

**FIGURE 1:**
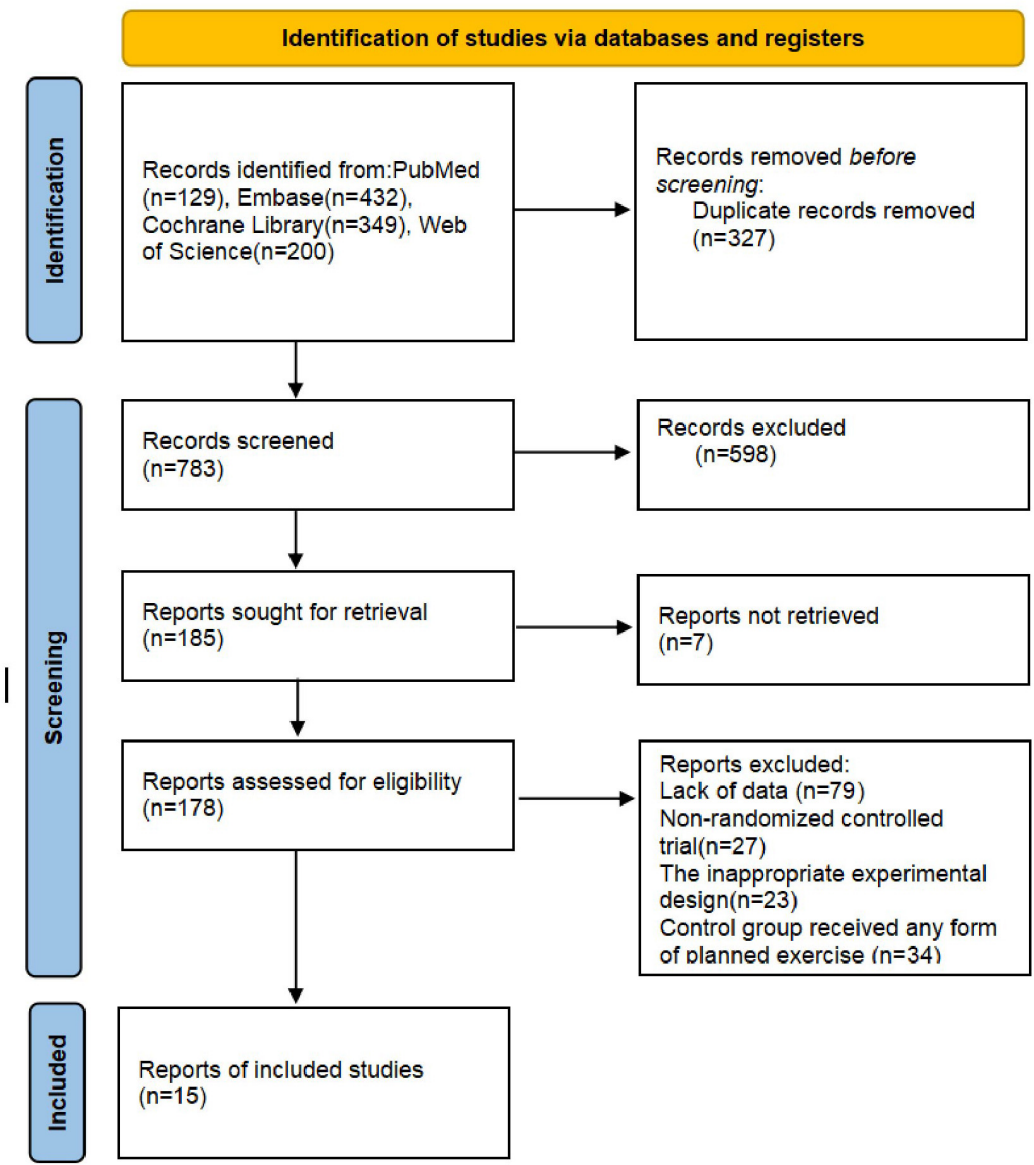
The PRISMA statement flow chart. Risk of bias assessment in studies.

According to the search strategy, a total of 1,110 studies were retrieved. After excluding 327 duplicate studies, the abstracts and titles of 783 studies were carefully reviewed. Following the exclusion of 598 titles and abstracts, along with 7 studies for which full-text articles could not be retrieved, 185 studies were read in full. Following full-text assessment, 163 studies were excluded for the following reasons: non-RCT (*n* = 27), lack of data (*n* = 79), inadequate trial design (*n* = 23), or any form of exercise in the control group (*n* = 34). Ultimately, 15 studies involving 1,699 patients were included in this meta-analysis. The included studies were multicenter trials originating from eight countries: Iran, Norway, China, Brazil, Turkey, Spain, South Korea, and the Netherlands. All were published in English. Single exercise sessions ranged from 15 to 60 min, predominantly lasting 30 or 60 min. Exercise frequency predominantly ranged from 2 to 3 sessions per week, with some studies employing daily or 5–7 sessions per week training regimens. Total intervention duration spanned 2–52 weeks, predominantly at 8, 12, and 24 weeks. All participants were individuals with axial spondyloarthritis. Primary outcome measures included disease activity (e.g., BASDAI, ASDAS), physical function (BASFI), spinal mobility (BASMI, thoracic expansion), and fatigue. The PRISMA statement flow chart (Figure 1 and Table 1).

**TABLE 1:** Basic characteristics of the included study.

Study	Country	Sample size (T/C)	Age (y), mean ± SD or median (range)		Duration/W		Intervention group	Intervention group supervision method	Intervention group implementation site	Control group	Main outcomes	Frequency	Post-intervention
Rahimibarghani et al. (39)	Iran	15, 15	35.9(6.4)	40.5(10.2)	7.9(8.3)	7.8(5.1)	Cardiorespiratory exercises and strength exercises	Home-based exercise program me (initial guidance and telephone reminders)	At home	Standard care	➀ ➁ ➂ ➃ ➄	60 min/time, 3 times/week, 4 weeks	Within 1 week
Sveaas et al. (25)	Norway	50.50	45.1(23–68)	47.2(24–69)	NA	NA	Cardiorespiratory exercises and strength exercises	Hospital supervision training	At the hospital	Standard care	➅	Cardiorespiratory exercises: 40 min/time, 3 times/week, 12 weeks; strength exercises: 20 min/time, 2 times/week, 12 weeks	12 weeks
Sveaas et al. (26)	Norway	50.50	45.1(23–68)	47.2(24–69)	NA	NA	Cardiorespiratory exercises and strength exercises	Hospital supervision training	At the hospital	Standard care	➀ ➁ ➂ ➃	Cardiorespiratory exercise: 40 min/time, 3 times/week, 12 weeks; strength exercises: 20 min/time, 2 times/week, 12 weeks	12 weeks
Xie et al. (27)	China	23.23	18-0 years old	18-60 years old	NA	NA	Cardiorespiratory exercises	1-4 weeks: hospital supervision training: 5-12 weeks: telephone reminders	At the hospital and at home	Standard care	➀ ➁	Cardiorespiratory exercises: ≥ 3 times/week, 15-20 min/time 12 weeks	12 weeks
Sveaas et al. (28)	Norway	50.50	45.1(23–68)	47.2(24–69)	NA	NA	Cardiorespiratory exercises and strength exercises	Hospital supervision training	At the hospital	Standard care	➀ ➁ ➂ ➃	Cardiorespiratory exercises: 3 times/week, 12 weeks; strength exercises: 2 times/week, 12 weeks	12 weeks
Sveaas et al. (29)	Norway	10.14	46.6 ± 13.6	49.9 ± 11.1	19.2 ± 19.8	28.6 ± 11.9	High-intensity cardiorespiratory exercises and strength exercises	Hospital supervision training	At the hospital	Standard care	➅	Cardiorespiratory exercises: 28-40 min/time, 3 times/week, 12 weeks; strength exercises: 20 min/time, 2 times/week, 12 weeks	12 weeks
Souza et al. (30)	Brazil	30.30	45(9.8)	43.8(10.2)	8.8(6.6)	9.6(7.8)	Strength exercises	Hospital supervision training	At the hospital	Standard care	➃	50 min/time, 2 times/week, 16 weeks	16 weeks
Karahan et al. (31)	Turkey	28.29	36.1 ± 12.4	36.6 ± 11.3	88.4 ± 51.2	91.2 ± 47.4	Cardiorespiratory exercises	Hospital supervision training	At the hospital	Standard care	➀ ➁	30 min/time, 5 times/week, 8 weeks	8 weeks
Sveaas et al. (32)	Norway	10.14	46.6(13.6)	49.9(11.1)	19.2(19.8)	28.6(11.9)	High-intensity cardiorespiratory exercises and strength exercises	Hospital supervision training	At the hospital	Standard care	➀ ➁ ➂ ➃	40-60 min/time, 3 times/week, 12 weeks	12 weeks
Rodríguez-Lozano et al. (33)	Spain	381.375	45 ± 12	46 ± 11	17 ± 10	18 ± 11	Cardiorespiratory exercises	Home-based exercise program me (initial guidance and telephone reminders)	At home	Standard care	➀ ➁	3-6 times/week, 24 weeks	24 weeks
Altan et al. (34)	Turkey	29.24	46.5(11.2)	43.6(10.1)	NA	NA	Cardiorespiratory exercises and strength exercises	Hospital supervision training	At the hospital	Standard care	➀ ➁ ➂ ➄	60 min/time, 3 times/week, 12 weeks	12 weeks
Ince et al. (35)	Turkey	15.15	33.67 ± 5.15	36.13 ± 7.20	8.27 ± 5.71	9.79 ± 6.46	High-intensity cardiorespiratory exercises and Strength exercises	Hospital supervision training	At the hospital	Standard care	➄	50 min/time, 3 times/week, 12 weeks	12 weeks
Lim et al. (36)	Korea	25.25	28.8 ± 9.3	28.1 ± 7.5	9.2 ± 0.76	8.6 ± 0.72	Cardiorespiratory exercises and strength exercises	Home-based exercise program me (initial guidance and telephone reminders)	At home	Standard care	➀	30 min/time, 7 times/week, 8 weeks	8 weeks
Sveaas et al. (29)	Norway	10.14	46.6 ± 13.6	49.9 ± 11.1	19.2 ± 19.8	28.6 ± 11.9	High-intensity cardiorespiratory exercises and strength exercises	Hospital supervision training	At the hospital	Standard care	➅	Cardiorespiratory exercises: 28-40 min/time, 3 times/week, 12 weeks; strength exercises: 20 min/time, 2 times/week, 12 weeks	12 weeks
van Wissen et al. (37)	Netherlands	110.104	51.9(11.7)	52.4(12.1)	14.1(11.3) (*n* = 97)	16.1(14.8) (*n* = 91)	Cardiorespiratory exercises and strength exercises	Hospital supervision training	At the hospital	Standard care	➀	2 times/week, ≤ 12 weeks, 1 time/week, ≥ 12 weeks	52 weeks
Acar et al. (38)	Turkey	28.27	44.14(8.03)	45.33(7.24)	13.93(7.80)	13.63(7.40)	Cardiorespiratory exercises and strength exercises	Digital supervision exercise	At home	Standard care	➀ ➁ ➂	3 times/week, 8 weeks	8 weeks

Outcome indicators: ➀ BASFI, ➁ BASDAI, ➂ BASMI, ➃ ASDAS, ➄ Chest Expansion, and ➅ Fatigue.

### 3.2 Assessment of bias risk

**FIGURE 2:**
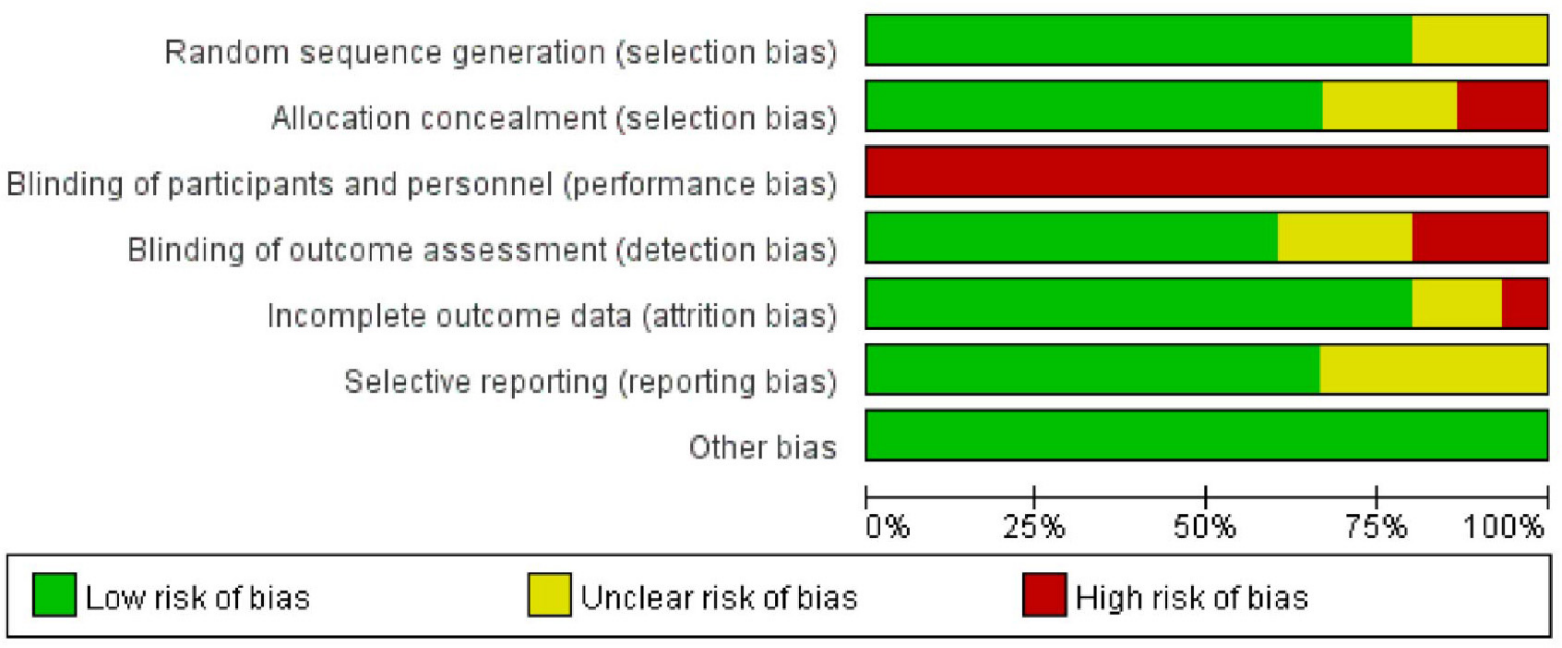
Risk of bias assessment in studies.

The quality of the included studies was moderate. Fourteen studies employed a two-arm design (25–38), whilst one study utilized a three-arm design (39). Twelve studies (25–30, 32–34, 37–39) reported appropriate randomization methods (random number tables or opaque sealed envelope allocation), presenting a low risk of bias. Three trials (31, 35, 36) did not explicitly describe randomization methods. Due to the specific nature of the interventions, no studies mentioned patient blinding. Ten trials (25–30, 32, 33, 37, 38) reported allocation concealment. Nine trials (25, 27, 28, 30, 32, 34, 36, 38, 39) reported blinding for outcome assessment. One study (27) had incomplete outcome data and a high risk of bias. Five studies (31, 33–36) exhibited selective reporting; other risk of bias factors were unclear (Figure 2 and Supplementary Figure 1).

### 3.3 Primary outcomes

#### 3.3.1 BASFI

**FIGURE 3:**
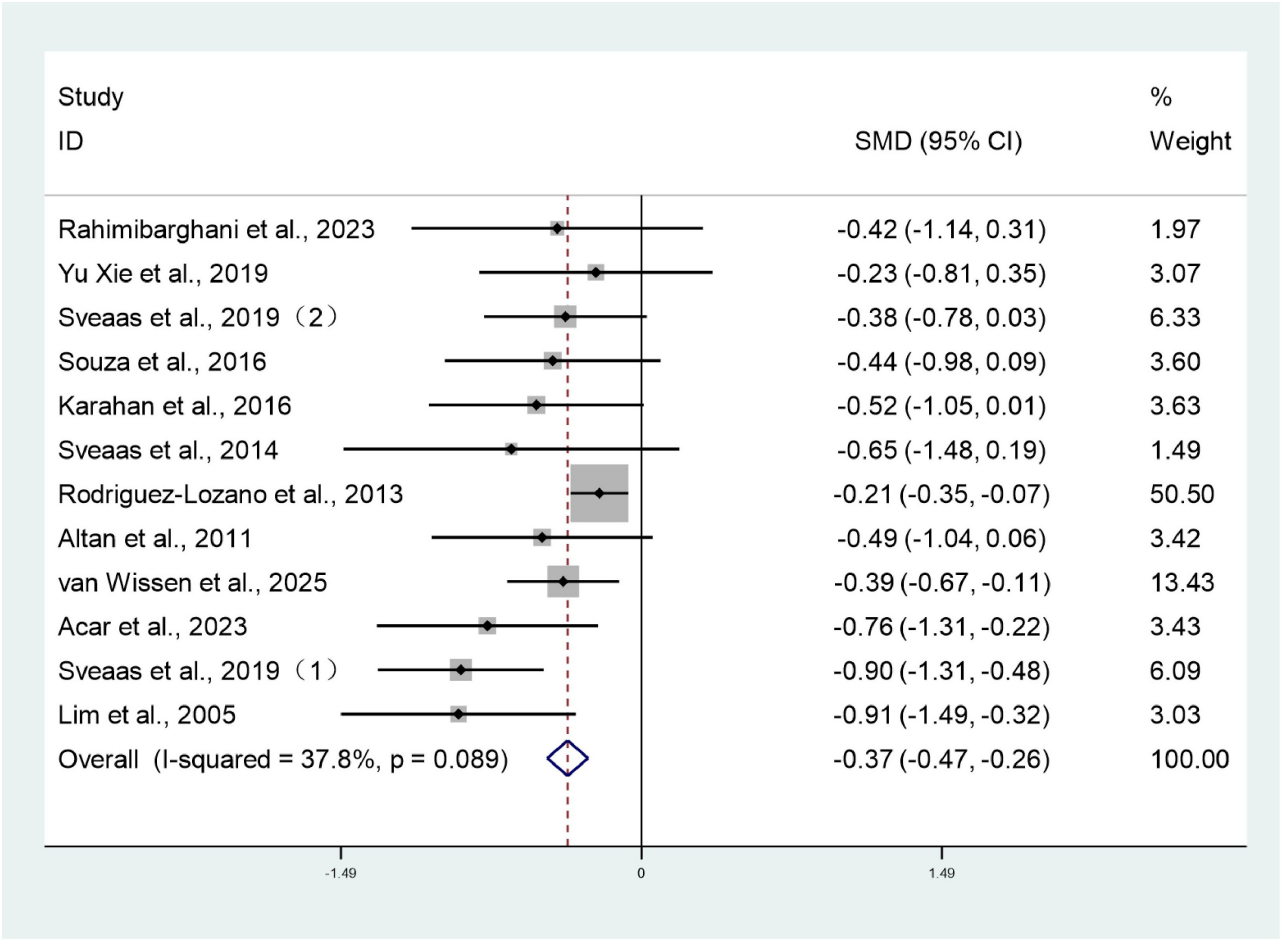
Forest plots of meta-analysis of BASFI.

Twelve studies (26–28, 30–34, 36–39) involving 1,545 patients reported BASFI scores for both intervention and control groups. Heterogeneity testing revealed *I*^2^ = 37.8%, indicating low heterogeneity, and analysis employed a fixed-effect model. The meta-analysis revealed that the BASFI scores in the treatment group were significantly lower than those in the control group (SMD: –0.37; 95% CI: –0.47, –0.26; *I*^2^ = 37.8%), with the difference being statistically significant (*P* < 0.05) (Figure 3).

#### 3.3.2 BASDAI

**FIGURE 4:**
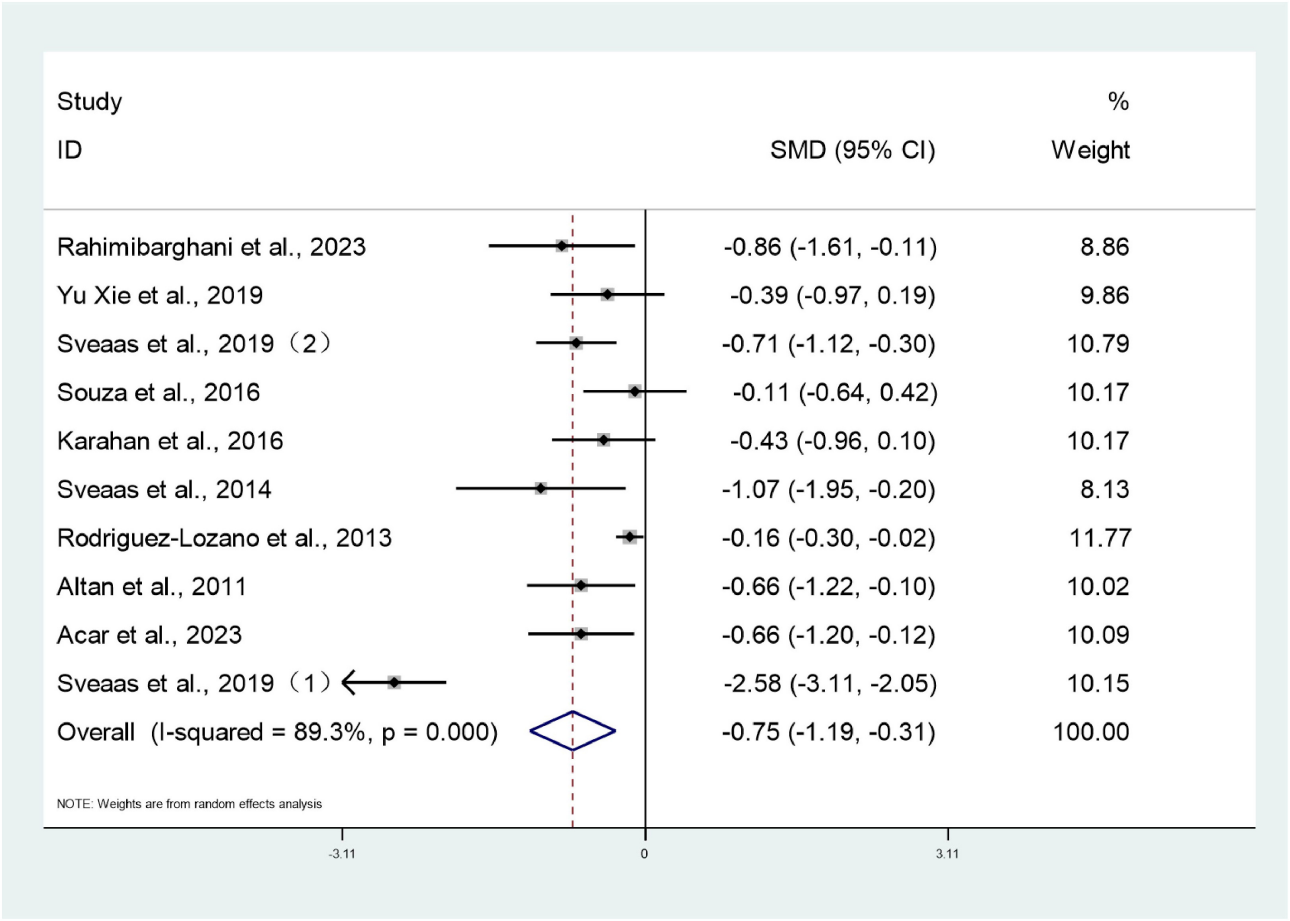
Forest plots of meta-analysis of BASDAI.

Ten studies (26–28, 30–34, 38, 39) involving 1,281 patients reported BASDAI scores for both intervention and control groups. Heterogeneity testing revealed an *I*^2^ value of 89.3%, indicating substantial heterogeneity, necessitating analysis using a random-effects model. The meta-analysis revealed that the BASDAI score in the trial group was significantly lower than that in the control group (SMD: –0.75; 95% CI: –1.19, –0.31; *I*^2^ = 89.3%), with the difference being statistically significant (*P* < 0.05) (Figure 4).

#### 3.3.3 BASMI

**FIGURE 5:**
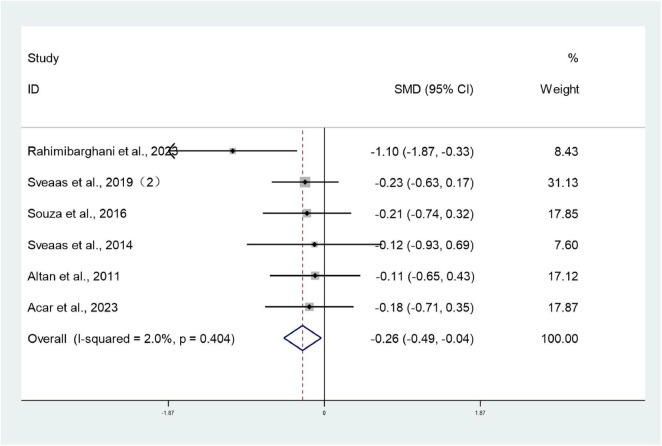
Forest plots of meta-analysis of BASMI.

Six studies (28, 30, 32, 34, 38, 39) involving 322 patients reported BASMI scores for both intervention and control groups. Heterogeneity testing revealed *I*^2^ = 2%, indicating minimal heterogeneity, and analysis employed a fixed-effect model. The meta-analysis revealed that the BASMI scores in the intervention group were significantly lower than those in the control group (SMD: –0.26; 95% CI: –0.49, –0.04; *I*^2^ = 2%), with the difference being statistically significant (*P* < 0.05) (Figure 5).

#### 3.3.4 ASDAS

**FIGURE 6:**
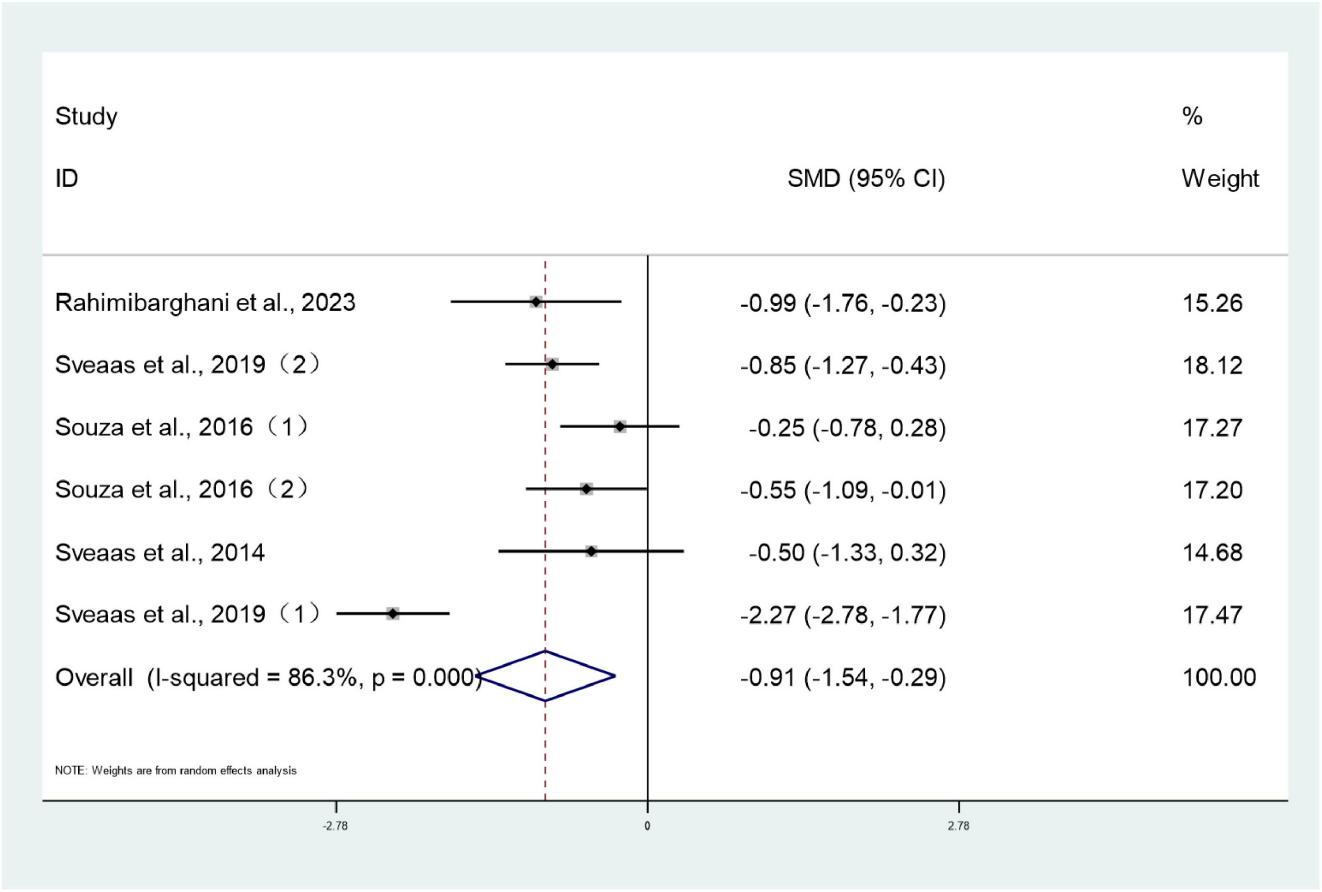
Forest plots of meta-analysis of ASDAS.

Six studies (26, 28, 30, 32, 39) involving 314 patients reported ASDAS scores for both treatment and control groups. Heterogeneity testing revealed an *I*^2^ value of 86.3%, indicating substantial heterogeneity, necessitating analysis using a random-effects model. The meta-analysis revealed that the ASDAS scores in the intervention group were significantly lower than those in the control group (SMD: –0.91; 95% CI: –1.54 to –0.29; *I*^2^ = 86.3%), with the difference being statistically significant (*P* < 0.05) (Figure 6).

#### 3.3.5 Thoracic expansion capacity

**FIGURE 7:**
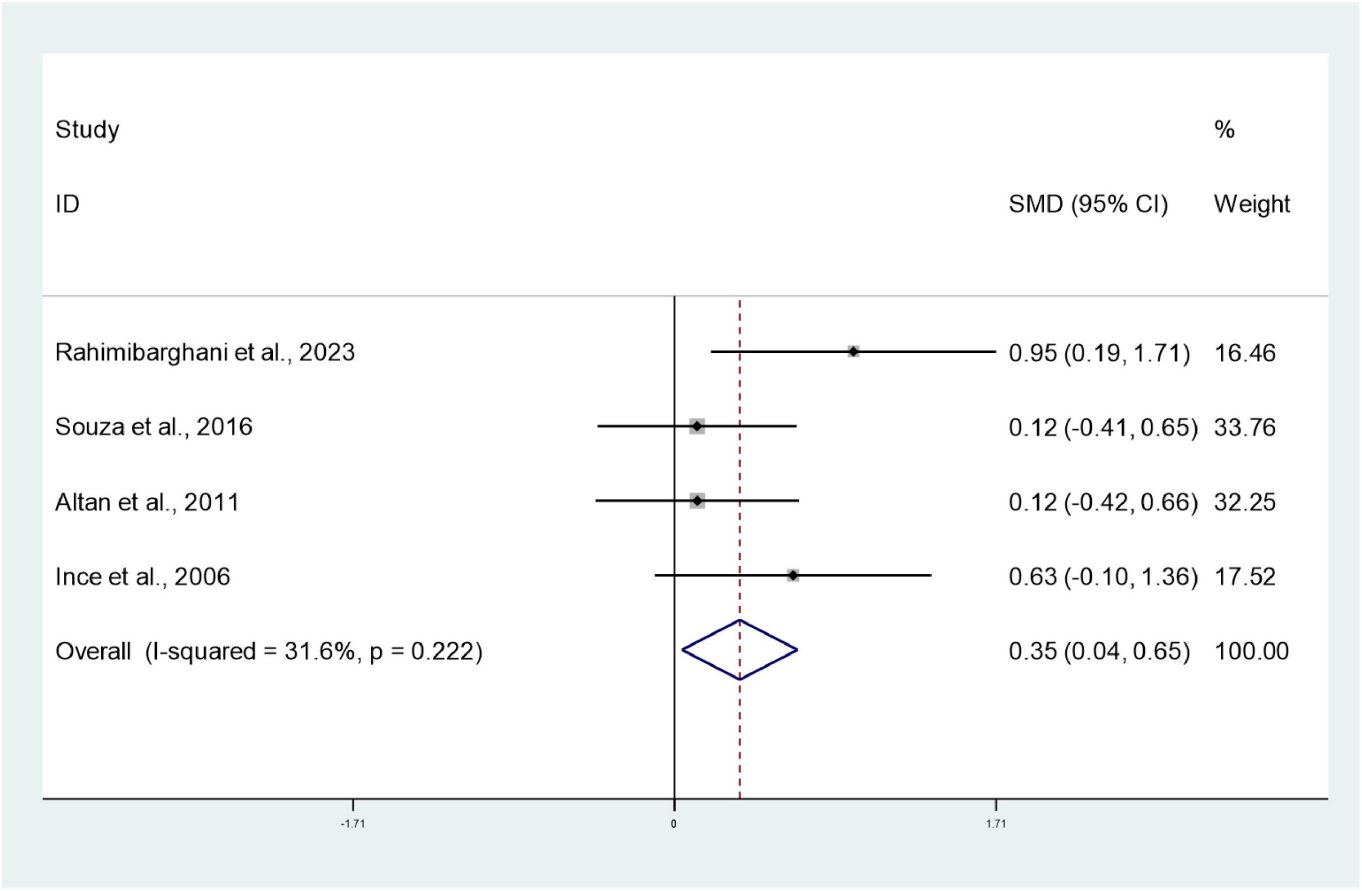
Forest plots of meta-analysis of thoracic expansion capacity.

Four studies (30, 34, 35, 39) involving 173 patients reported thoracic expansion capacity in the intervention and control groups. Heterogeneity testing revealed *I*^2^ = 31.6%, indicating low heterogeneity, and a fixed-effect model was employed for analysis. The meta-analysis revealed significantly greater thoracic expansion capacity in the intervention group compared to the control group (SMD: 0.35; 95% CI: 0.04, 0.65; *I*^2^ = 31.6%), with the difference being statistically significant (*P* < 0.05) (Figure 7).

#### 3.3.6 Fatigue

**FIGURE 8:**
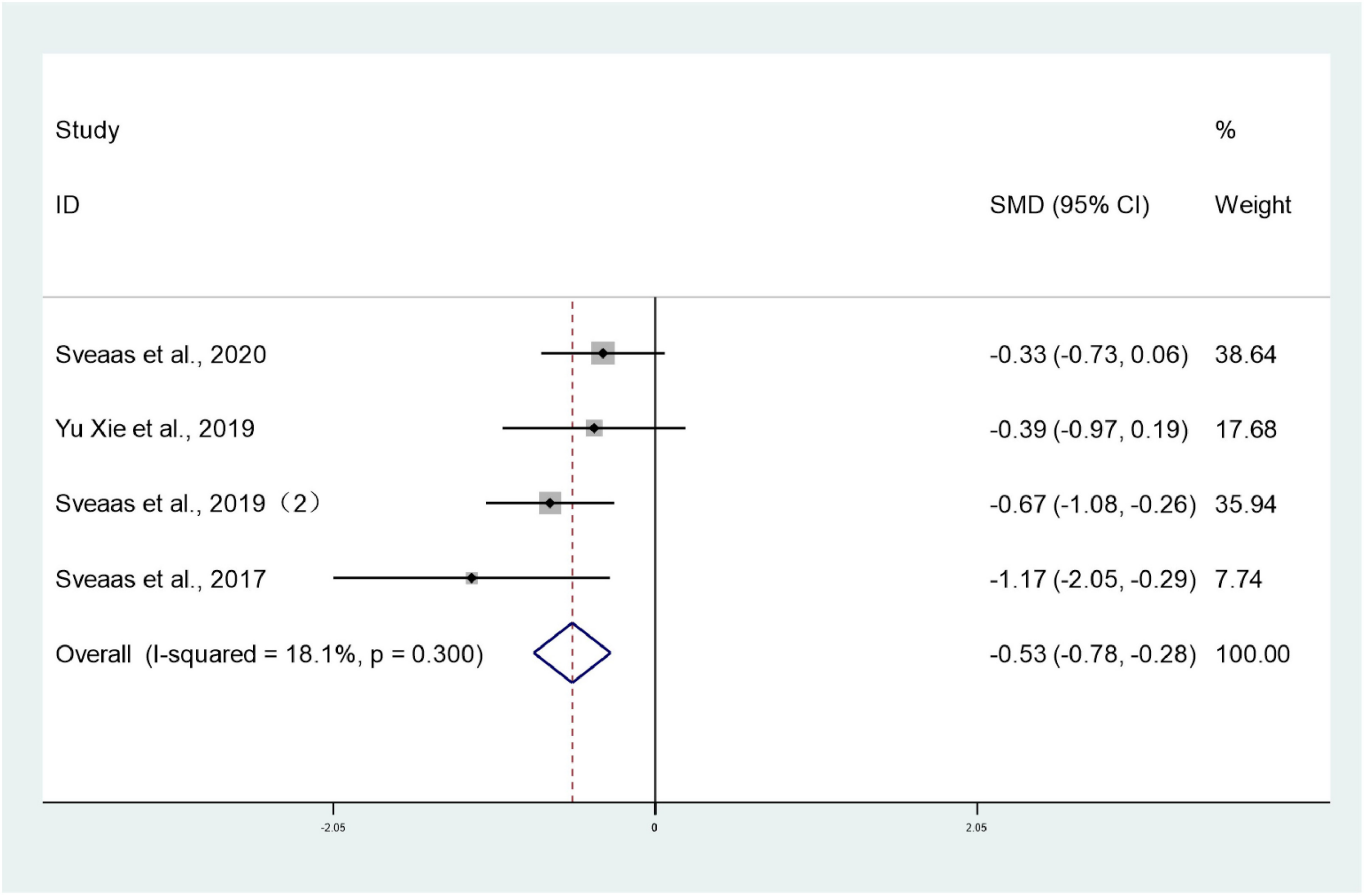
Forest plots of meta-analysis of fatigue.

Four studies (25, 27–29) involving 270 patients reported fatigue levels in both intervention and control groups. Heterogeneity testing revealed *I*^2^ = 18.1%, indicating low heterogeneity, and analysis employed a fixed-effect model. The meta-analysis revealed significantly lower fatigue levels in the intervention group compared to the control group (SMD: –0.53; 95% CI: –0.78 to 0.28; *I*^2^ = 18.1%), with the difference being statistically significant (*P* < 0.05) (Figure 8).

### 3.4 Subgroup analysis

#### 3.4.1 BASDAI subgroup analysis

Given the heterogeneity observed in the meta-analysis of BASDAI scores, a subgroup analysis was conducted based on exercise intervention type to investigate sources of heterogeneity. Studies were categorized into low-intensity mind-body exercises and high-intensity equipment training.

Results indicated that within the low-intensity mind-body exercise group, four studies (28, 34, 38, 39) reported BASDAI scores for both intervention and control groups. Heterogeneity testing revealed *I*^2^ = 0%, suggesting minimal heterogeneity, thus employing a random-effects model for analysis. The meta-analysis revealed that the experimental group exhibited significantly lower BASDAI scores than the control group (SMD: –0.70; 95% CI: –0.97 to –0.44; *I*^2^ = 0%), with the difference being statistically significant (*P* < 0.05).

**FIGURE 9:**
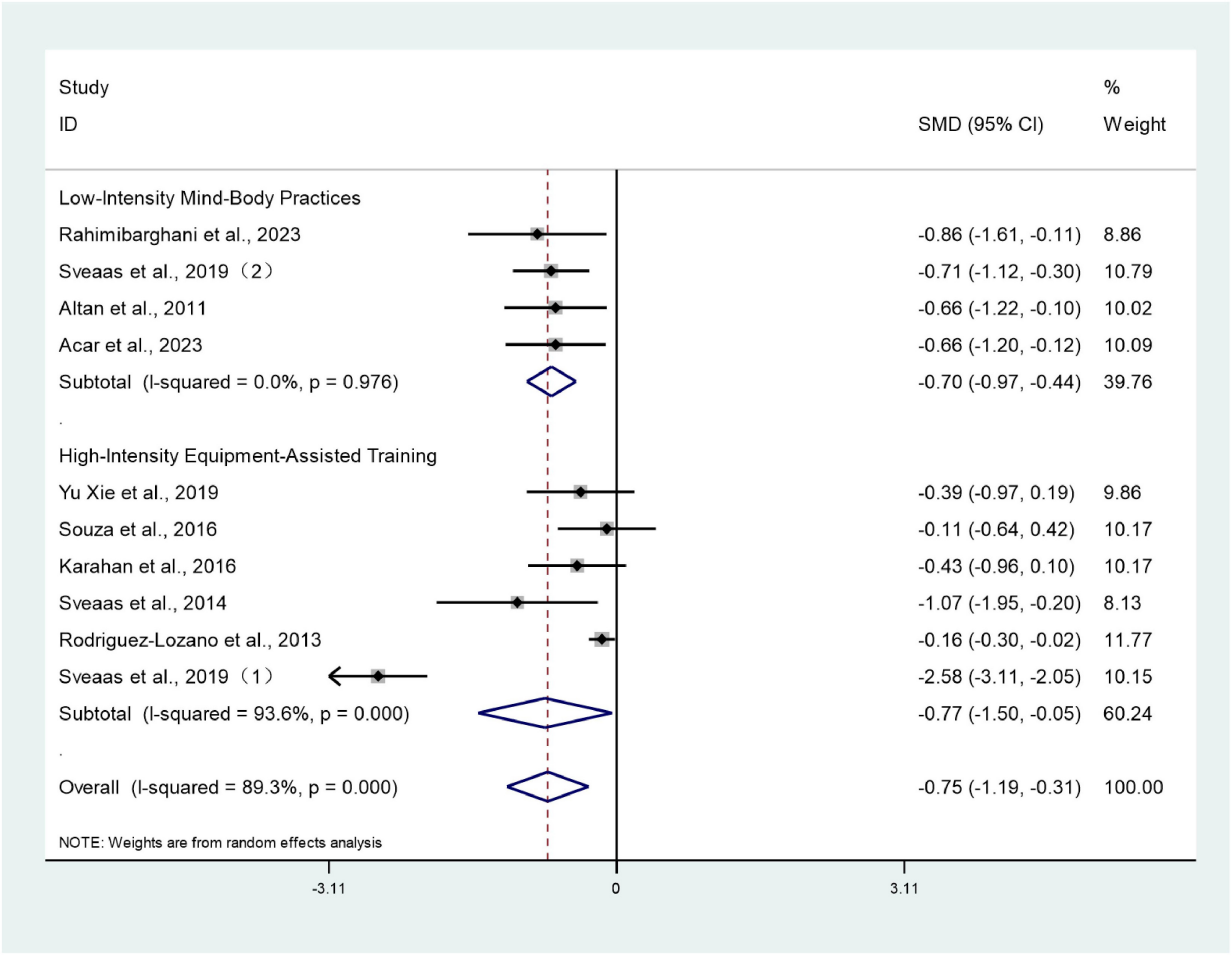
Forest plots of meta-analysis of BASDAI subgroup analysis.

Within the high-intensity equipment training group, exercise therapy also significantly improved BASDAI scores. Six studies (26, 27, 30–33) reported BASDAI scores for both the intervention and control groups. Meta-analysis revealed significantly lower BASDAI scores in the intervention group compared to the control group (SMD = –0.77, 95% CI: –1.50 to –0.05; I^2^ = 93.6%), with a statistically significant difference (*P* < 0.05). Although all six studies were classified as high-intensity, Sveaas et al.’s (26) intervention model featured markedly greater cardiopulmonary metabolic load and a more systematized training approach, resulting in a markedly different effect size compared to other studies. This may represent the primary source of heterogeneity (Figure 9).

Clinical significance of subgroup analysis: Low-intensity mind-body exercises (such as Baduanjin and yoga) demonstrate stable efficacy in improving disease activity (*I*^2^ = 0%) and offer greater safety, making them more suitable for axSpA patients experiencing significant pain, severe functional limitations, or elderly individuals. High-intensity equipment training yields slightly higher effect sizes but exhibits substantial heterogeneity, potentially related to variations in training intensity control and patient fitness levels, and is more appropriate for younger patients with better baseline physical fitness. Exercise prescription should be tailored to individual patient circumstances.

#### 3.4.2 ASDAS subgroup **analysis**

Given the heterogeneity observed in the meta-analysis of ASDAS, a subgroup analysis was conducted to investigate the sources of heterogeneity by grouping studies according to intervention duration (≤ 12 weeks versus 16 weeks).

Results indicated that among studies with a 16-week intervention duration, two studies (30) reported ASDAS scores for both intervention and control groups. Meta-analysis revealed significantly lower ASDAS scores in the intervention group compared to the control group (SMD: –0.39; 95% CI: –0.77 to –0.02; *I*^2^ = 0%), with the difference being statistically significant (*P* < 0.05).

**FIGURE 10:**
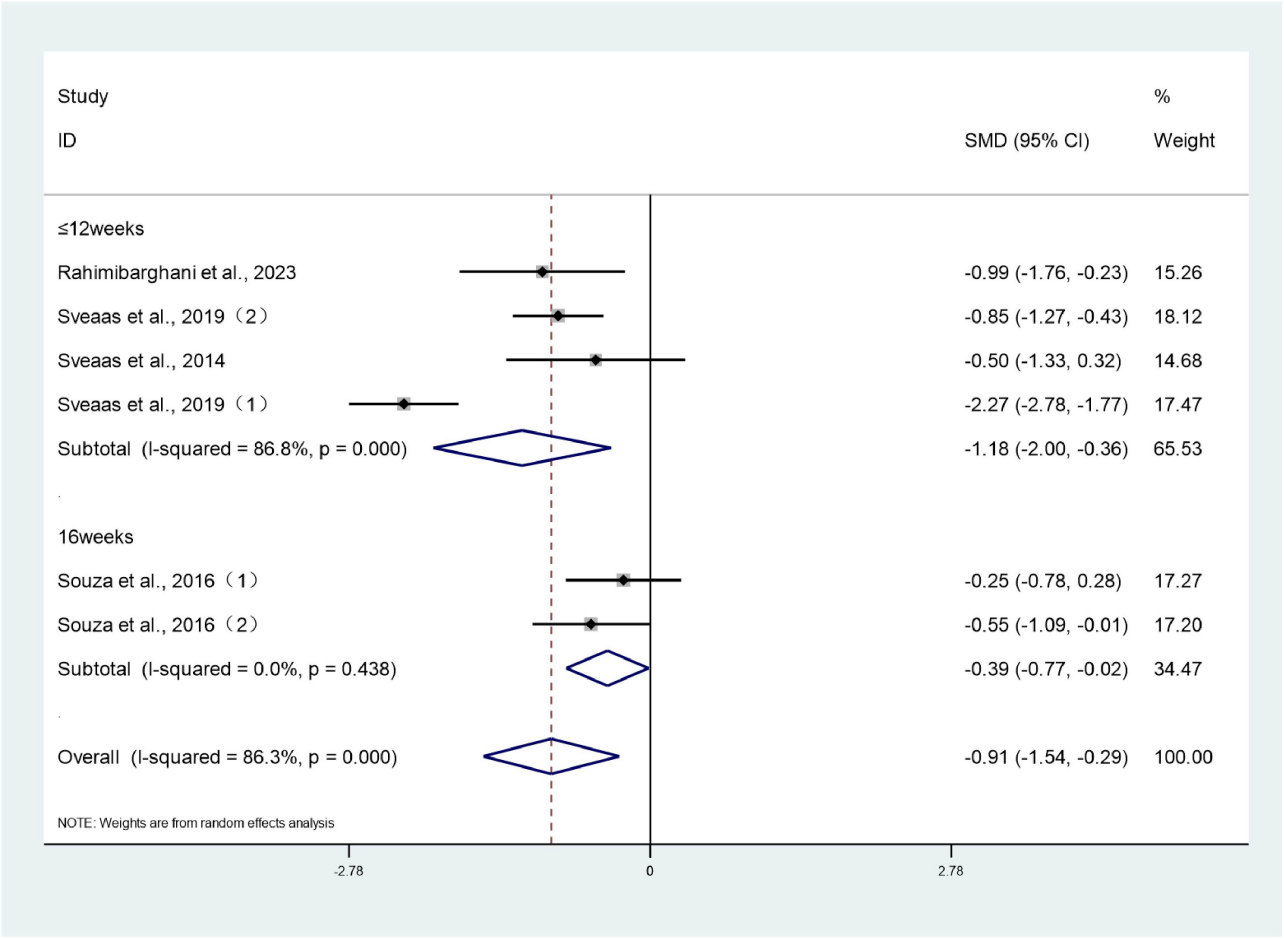
Forest plots of meta-analysis of ASDAS subgroup analysis.

Among studies with ≤ 12 weeks’ intervention duration, four studies (26, 28, 32, 39) reported ASDAS scores for both intervention and control groups. Meta-analysis indicated significantly lower ASDAS scores in the intervention group compared to the control group (SMD: –1.18; 95% CI: –2.00 to –0.36; *I*^2^ = 86.8%), with the difference being statistically significant (*P* < 0.05). Although all four studies had intervention durations ≤ 12 weeks, Sveaas et al.’s (26) intervention protocol was markedly more intensive in terms of cardiopulmonary metabolic load and featured a more systematic training approach, resulting in a larger effect size. This may represent the primary source of heterogeneity (Figure 10).

Clinical significance of subgroup analysis: Short-term intervention (≤12 weeks) rapidly reduces disease activity (ASDAS SMD = –1.18), serving as an adjunctive treatment during acute disease flare-ups. Long-term intervention (16 weeks) yields moderate yet stable effects, making it suitable for maintenance therapy during disease remission. In clinical practice, intervention duration may be adjusted according to the patient’s disease stage.

### 3.5 Sensitivity analysis

We conducted a sensitivity analysis to assess the robustness of the results for six key indicators. Excluding any single study had no significant impact on the magnitude of the pooled effect estimates. The standardized mean difference (SMD) values differed only marginally from the original overall SMD values, and statistical significance was maintained, with *p*-values consistently below 0.05. This indicates that the meta-analysis results for the aforementioned six indicators are robust and reliable (Supplementary Figures 2-7).

### 3.6 Publication bias

**FIGURE 11:**
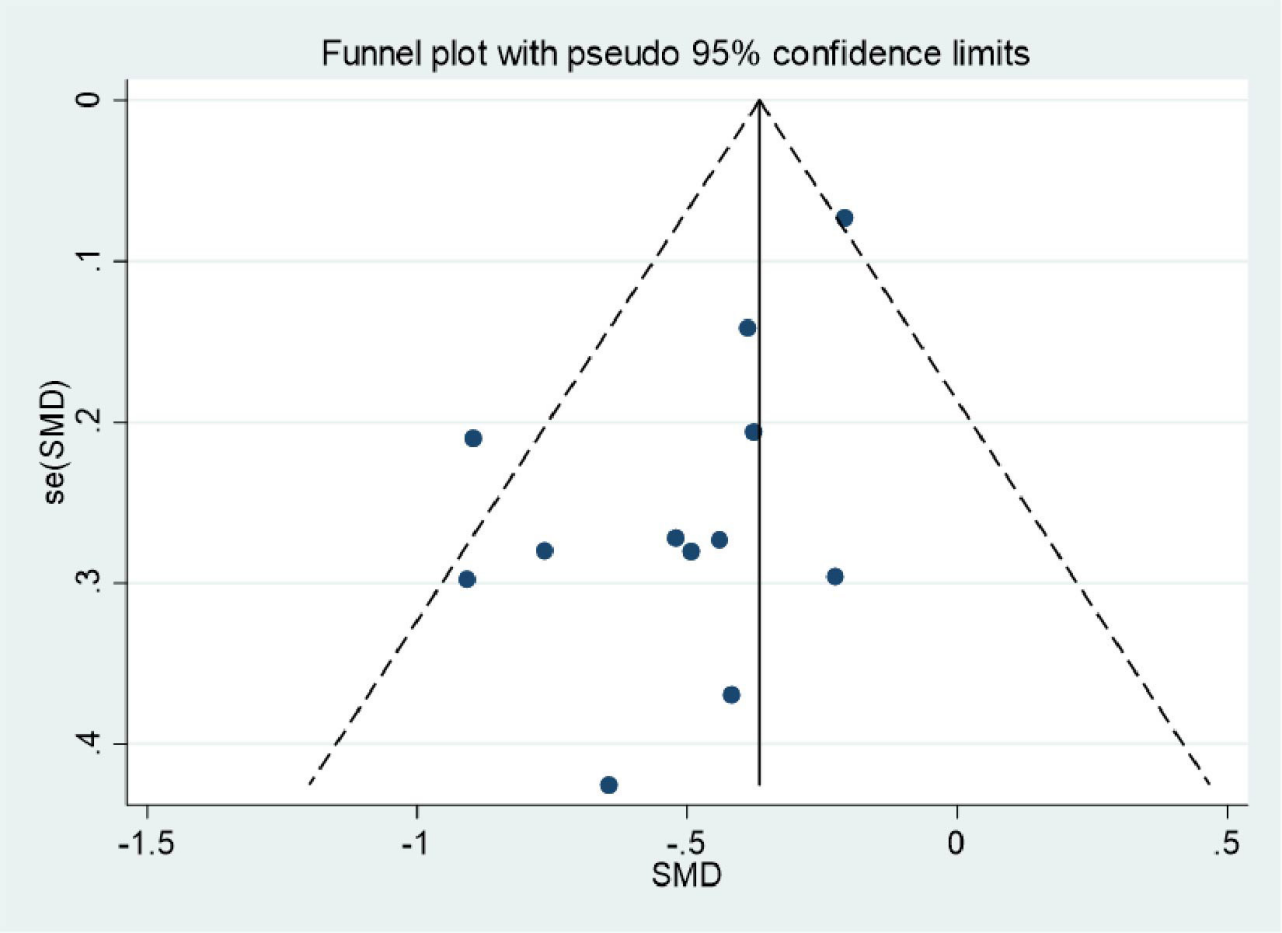
Funnel plots of meta-analysis of BASFI.

**FIGURE 12:**
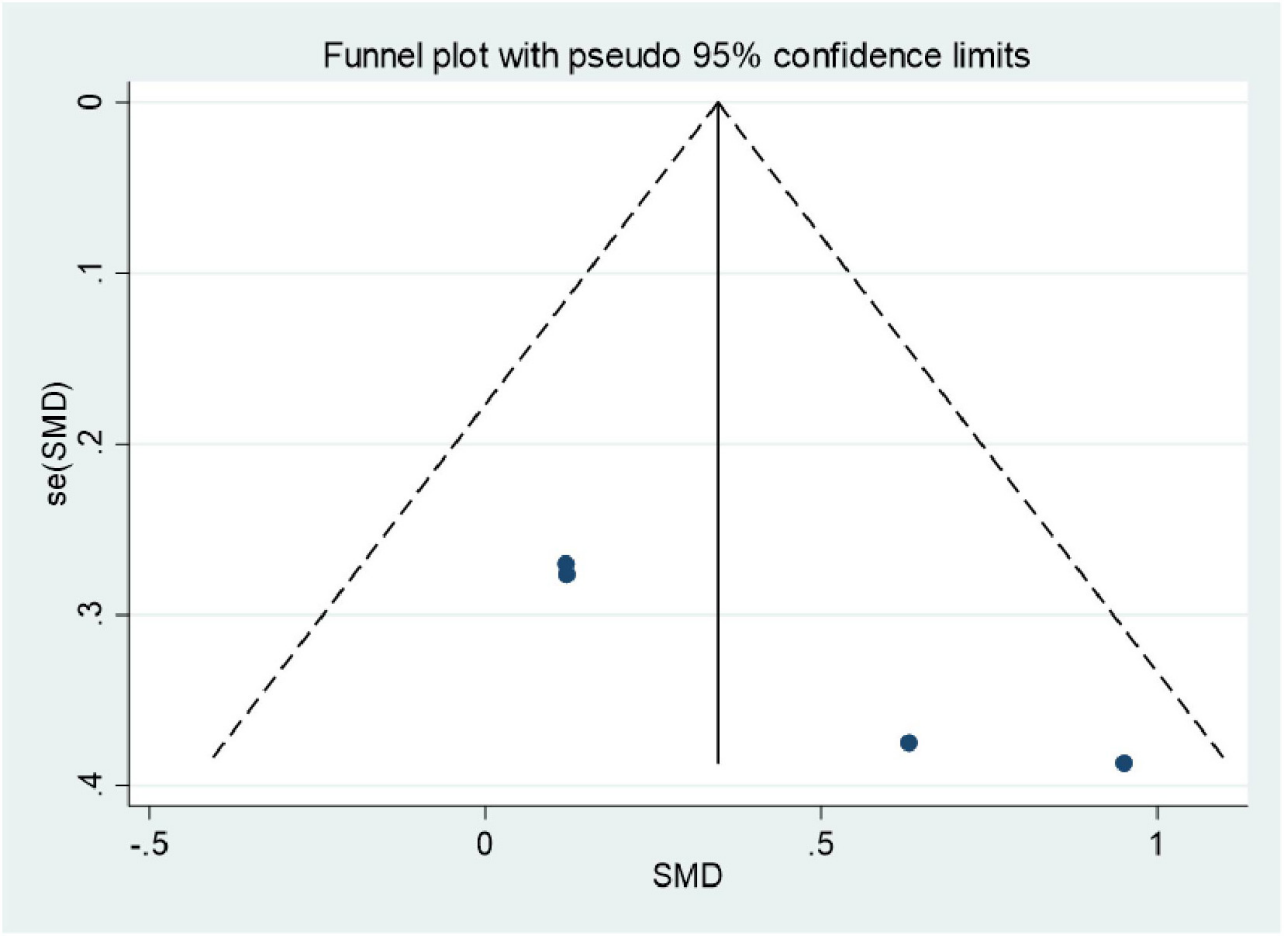
Funnel plots of meta-analysis of thoracic expansion capacity.

Publication bias was assessed using funnel plots. Plots were generated for improvements in BASFI and thoracic expansion following exercise. The results revealed asymmetry in the funnel plots. The Egger’s test values were 0.011 for BASFI and 0.025 for thoracic expansion. Consequently, the included studies exhibited a degree of publication bias (Figures 11, 12).

### 3.7 Certainty assessment

The certainty of evidence was assessed using the GRADE approach, which systematically evaluates and may downgrade the quality of evidence based on several domains: risk of bias, inconsistency, indirectness, imprecision, and publication bias. The specific reasons for the ratings across the different outcome measures are detailed as follows to enhance transparency:

Chest Expansion: The evidence was rated as very low quality. This rating results from significant concerns across multiple domains: a serious risk of bias within the included studies, serious imprecision in the effect estimates (as indicated by a wide confidence interval that may cross the threshold for clinical importance), and suspected publication bias.

BASFI: The evidence was rated as low quality. The primary reasons for downgrading were a serious risk of bias in the study methodologies and potential publication bias.

Fatigue: The evidence was similarly rated as low quality. This assessment was based on a serious risk of bias and serious imprecision in the results.

BASDAI and ASDAS: The evidence for both measures was rated as low quality. The downgrading was due to a serious risk of bias and important inconsistency among the study results (significant heterogeneity or variability in the estimated effects).

BASMI: The evidence was rated as moderate quality, representing the highest quality among the outcomes assessed. It was downgraded by one level solely due to a serious risk of bias; no other limitations (inconsistency, indirectness, imprecision) were identified.

This structured explanation clarifies the specific GRADE domains that influenced each quality rating, thereby addressing the ambiguity and strengthening the interpretation of the evidence certainty (Supplementary Figures 1).

## 4 Discussion

This meta-analysis, incorporating 15 randomized controlled trials involving 1,699 patients, provides robust evidence for the efficacy of exercise interventions as a core non-pharmacological treatment strategy for axial spondyloarthritis (axSpA). Our findings indicate that exercise prescription yields statistically significant and clinically meaningful improvements across multiple key outcome measures, including disease activity, physical function, spinal mobility, thoracic expansion, and fatigue levels. The following discussion interprets these results in light of prior research, explores potential mechanisms of action, outlines clinical implications, acknowledges study limitations, and proposes directions for future research.

### 4.1 Comparison with previous studies

The findings of this study are broadly consistent with previous systematic reviews and meta-analyses in this field, while expanding their scope and depth. Early research primarily confirmed the positive effects of exercise on disease activity (e.g., BASDAI) and functional status (e.g., BASFI) (40). This analysis not only reaffirms these core benefits—demonstrating significant reductions in BASDAI (SMD = –0.75) and BASFI (SMD = –0.37)—but also provides quantitative evidence of exercise’s positive impact on several previously under-examined outcome measures. Notably, compared with the study by Zhang et al. (41), the significant improvement in thoracic expansion (SMD = 0.35) is particularly noteworthy, as it addresses a core pathophysiological component of axSpA involving costovertebral and costothoracic joints, which leads to restrictive lung disease. Moreover, the significant improvement in fatigue levels (SMD = –0.53) highlights exercise’s role in managing this frequently overlooked debilitating symptom, thereby providing a more comprehensive assessment of patient health status. The magnitude of ASDAS improvement observed in this study (SMD = –0.91) appears superior to findings from some prior meta-analyses. For instance, Zhang et al. (41) reported a weighted mean difference (WMD) of –0.44 (95% CI, –0.64 to –0.24) for exercise interventions on ASDAS. Although direct numerical comparisons between WMD and SMD are limited, the SMD value of –0.91 falls within the “large effect size” category according to Cohen’s criteria. We believe that ASDAS demonstrated a more pronounced improvement effect in this study, potentially attributable to several factors: Firstly, the ASDAS assessment is more comprehensive, integrating subjective symptoms (pain, stiffness) with objective indicators (CRP/ESR), whereas the BASDAI relies primarily on patient self-reporting. Given that most interventions in this study could not be conducted under blinding conditions (due to the specific nature of exercise interventions), this may have led to greater subjective bias in the BASDAI. The objective components of the ASDAS partially offset this bias. Secondly, exerciseyt anti-inflammatory effects manifest not only through subjective symptom relief but also by modulating immune-inflammatory pathways to reduce CRP levels (42–44). This aligns more closely with ASDAS’s objective metric assessment, thereby yielding more pronounced improvement effects. Furthermore, certain high-intensity exercise programs included in the studies demonstrated more marked suppression inflammatory factors, further amplifying the improvement observed in ASDAS scores. This suggests that the exercise interventions included in our analysis may yield more pronounced clinical benefits. This may be attributed to our inclusion of recent RCTs employing more intensive and structured exercise programs combining high-intensity cardiopulmonary training with strength training. Our subgroup analyses further deepened these comparisons by revealing that exercise effects may be influenced by its form and duration of intervention.

### 4.2 Interpretation of results and mechanism analysis

The results of this meta-analysis consistently demonstrate that exercise interventions significantly improve disease activity, physical function, spinal mobility, thoracic expansion, and fatigue symptoms in axSpA. These benefits are not isolated but stem from exercise’s multi-targeted, multi-level regulation of core pathophysiological pathways in axSpA. The following sections provide an in-depth explanation of potential mechanisms based on the study findings.

#### 4.2.1 Immunomodulation and anti-inflammatory effects: the core basis for disease activity improvement

The observed significant reduction in disease activity (as measured by BASDAI and ASDAS) fundamentally stems from exercise-induced systemic anti-inflammatory effects. This aligns closely with axSpA’s nature as an autoinflammatory disorder. Regular moderate-intensity exercise promotes the release of myokines such as interleukin-6 (IL-6) from muscle tissue. During the acute phase of exercise, IL-stimulates the production of cortisol in the adrenal cortex and C-reactive protein (CRP) in hepatocytes (42). More importantly, it induces the generation of anti-inflammatory cytokines such as interleukin-10 (IL-10) (43). This anti-inflammatory environment may help suppress the pathogenic activity of Th17 cells, thereby reducing levels of TNF-α and IL-17 (44). Furthermore, exercise promotes the redistribution of immune cells (such as regulatory T cells, Tregs) within the body, enhancing their immunosuppressive functions (45). This holistic regulation of the “neuro-endocrine-immune” network constitutes the biological basis for exercise’s fundamental alleviation of core symptoms such as inflammatory low back pain and morning stiffness.

#### 4.2.2 Biomechanical improvement and structural-functional enhancement: key pathways for elevating physical function and spinal mobility

Improvements in BASFI and BASMI scores reveal exercise’s direct benefits at the musculoskeletal level. Chronic inflammation and pain in axSpA often lead to reduced activity, causing disuse atrophy in core muscles (e.g., transversus abdominis, multifidus) and postural abnormalities (e.g., spinal kyphosis), which further exacerbate joint loading and functional impairment (46, 47). Targeted strength and flexibility training can disrupt this vicious cycle through dual mechanisms: Strength training for core and paraspinal muscles provides the spine with a “muscular ligament,” effectively sharing mechanical stress on the spine and sacroiliac joints, improving postural control, thereby reducing pain and enhancing capacity for daily activities like walking and bending (48–50); Sustained axial extension and rotational movements help maintain flexibility in the intervertebral and costovertebral joints, counteracting fibrosis and osteoarthritic tendencies potentially induced by inflammation. This directly improves spinal mobility as assessed by the BASMI (51).

#### 4.2.3 Cardiopulmonary function and respiratory mechanics optimization: specific mechanisms underlying thoracic expansion improvement

This study identified a significant increase in thoracic expansion, a finding of particular clinical significance. AxSpA can affect costovertebral and costal joints, leading to thoracic stiffness. Exercise intervention reverses this condition through two pathways: aerobic exercise and specific respiratory training enhance the strength and endurance of the diaphragm—the primary respiratory muscle—restoring its dominant role and improving ventilation efficiency (52, 53). Secondly, exercises such as chest expansion and stretching directly target the small thoracic vertebral joints and costovertebral joints, alleviating their stiffness and thereby physically increasing the range of motion of the thoracic cage (54). Improved thoracic expansion signifies not merely enhanced assessment metrics, but substantive gains in respiratory function and quality of life for patients.

### 4.3 Clinical implications

The findings of this meta-analysis provide direct and practical guidance for the clinical management of axial spondyloarthritis (axSpA). Exercise should be regarded as a core therapeutic component of equal importance to pharmacological treatment. Considering the methods of supervision, feasibility, and the positioning of digital interventions, the specific recommendations are as follows:

#### 4.3.1 Positioning and feasibility analysis of hospital-supervised exercise

Eleven studies examined hospital-supervised exercise (25–32, 34, 35, 37). This training model represents the preferred intervention for “patients in the acute phase and those with high disease activity,” demonstrating significant therapeutic advantages. This stems from professional guidance ensuring appropriate exercise intensity and movement technique, alongside timely program adjustments to mitigate injury risks. However, widespread implementation of hospital-supervised exercise for “the majority of axSpA patients” faces feasibility challenges: Resource and geographical constraints: Insufficient healthcare resources in developing countries or remote areas may hinder large-scale hospital-supervised programs. Patient adherence limitations: As axSpA predominantly affects young and middle-aged adults (1), balancing work commitments with treatment may reduce long-term adherence due to the time cost of regular hospital visits. Adaptability for special populations: Elderly patients or those with mobility impairments (e.g., severe spinal deformities) cannot participate in hospital-based exercise. Feasible solution: Implement a “stepwise intervention approach”—During disease flare-ups (BASDAI ≥ 5), employ hospital-supervised exercise (8–12 weeks) for rapid symptom control; During remission (BASDAI < 3.5), transition to digitally supervised or home-based independent exercise to maintain efficacy, balancing therapeutic outcomes with accessibility.

#### 4.3.2 Positioning and value of digital interventions

One study on digital interventions (38) positions this training as a “critical adjunct” to hospital-supervised exercise, with core value in addressing “accessibility and long-term adherence” challenges. Applicable scenarios: Serves patients in medically underserved areas, those with demanding work schedules preventing hospital visits, and those requiring maintenance therapy during remission. Limitations: Dependent on internet connectivity; operational challenges for elderly or less educated patients; absence of on-site correction for complex movements (e.g., strength training, spinal extension) may compromise efficacy. Future positioning: Should function as a “long-term maintenance tool” for axSpA exercise therapy, integrating real-time app monitoring (e.g., heart rate, exercise duration) to optimize intervention parameters, while incorporating online interactive modules (e.g., remote correction by rehabilitation therapists) to enhance efficacy.

#### 4.3.3 Correlation between digital interventions and outcome measures

Digital interventions offer “targeted advantages” in improving outcome measures: Superior long-term outcomes: The flexibility of digital interventions enhances patient adherence over extended periods, with sustained engagement facilitating greater functional recovery; Alignment with subjective outcome measures: Features like daily check-ins and symptom logging enable real-time monitoring of fatigue levels and pain scores, allowing patients to visibly track improvements and further boost compliance; Evidence quality limitations: Currently, only one study (38) exists on digital interventions, with GRADE evidence quality rated as low (due to risk of bias and sample size constraints). Future research requires larger sample sizes and optimized digital tools (e.g., incorporating sensors to monitor movement compliance) to enhance the magnitude of outcome improvements and evidence reliability.

However, it should be clarified that although the exercise effects for all outcome measures were statistically significant, the evidence for most measures had low confidence levels. Therefore, over-reliance on effect estimates should be avoided in clinical practice.

### 4.4 Limitations and future directions

Although this study rigorously synthesized the highest-level current evidence to confirm the comprehensive benefits of exercise interventions for axSpA patients, several aspects warrant refinement in future research. Firstly, included studies exhibited variations in specific exercise program parameters (e.g., intensity, frequency). While this clinical heterogeneity was explored via subgroup analyses, it may still impact the precision of pooled results. Secondly, owing to the nature of exercise interventions, most studies encountered difficulties in implementing blinding for participants and practitioners, potentially introducing bias in the assessment of subjective outcome measures such as BASDAI. Furthermore, this analysis primarily focused on short-to-medium-term efficacy (≤ 52 weeks); the long-term effects of exercise interventions on disease progression and structural changes warrant particular attention in future research. These limitations also point to directions for subsequent research, including conducting more high-quality, long-term follow-up randomized controlled trials and exploring personalized exercise prescriptions based on individual characteristics. The evidence for most outcome measures exhibits low confidence (low/very low quality), primarily influenced by risks of bias, heterogeneity, imprecision, and publication bias. This compromises the reliability of effect estimates, constituting one of the core limitations of this study. The present analysis primarily focuses on short-to-medium-term efficacy (≤ 52 weeks). The long-term effects of exercise interventions on disease progression and structural changes remain unclear, necessitating further validation of sustained efficacy.

### 4.5 The impact and interpretation of publication bias

This study identified significant publication bias in the exercise-induced improvements for BASFI (Egger’s *P* = 0.011) and thoracic expansion (Egger’s *P* = 0.025) through funnel plots and Egger’s test, with funnel plots exhibiting asymmetric distributions on both sides (Figures 11, 12). The presence of this bias necessitates a comprehensive interpretation considering both study design and evidence quality, thereby avoiding overinterpretation of the findings.

#### 4.5.1 Potential sources of publication bias

Small-sample positive studies are more likely to be published: Among the included BASFI-related studies, both small-sample studies (32, 39) (sample size ≤ 30 cases) reported significant improvements in mobility. Potential small-sample negative studies or those showing no significant effect were not retrieved, leading to an overestimation of the pooled effect size.

Differences in clinical focus of outcome measures: Thoracic expansion, as a secondary outcome measure for axSpA, has received limited attention in previous studies. Some researchers may be more inclined to submit positive results, whilst studies reporting negative findings may be rejected by journals or not submitted due to perceived “insufficient academic value.”

Inadequate inclusion of grey literature: This study only searched mainstream English databases and did not include grey literature such as unpublished research reports or conference abstracts, potentially omitting some negative results.

#### 4.5.2 Specific implications for research findings

Impact on BASFI: According to the GRADE assessment, the quality of evidence for BASFI was rated as low (the primary reasons for downgrading included risk of bias and publication bias). Publication bias may lead to an overestimation of the effect size for exercise on improvements in physical function. Clinicians should note that the actual improvement in daily functioning for axSpA patients may be slightly lower than the pooled effect size reported in this study (SMD = –0.37).

Impact on thoracic expansion: The evidence quality for thoracic expansion is very low (downgraded due to risk of bias, publication bias, and imprecision), with the pooled effect size (SMD = 0.35) being the least reliable. Given that only four studies (30, 34, 35, 39) related to thoracic expansion were included, with a total sample size of 168 patients, the small sample size combined with potential publication bias may have amplified the effect size. Clinical decision-making should therefore incorporate individual patient respiratory function assessments and should not rely solely on the findings of this study.

## 5 Conclusion

This systematic review and meta-analysis provides high-level evidence that prescribed exercise is an effective intervention for improving multiple health domains in patients with axial spondyloarthritis (axSpA). It effectively reduces disease activity and fatigue while enhancing physical function, spinal mobility, and thoracic expansion. Findings underscore the imperative for clinicians to actively advocate for and integrate structured, individualized exercise programs into the standard care pathway for every axSpA patient. Future research should focus on optimizing prescription parameters and confirming the long-term benefits of exercise as a cornerstone of axSpA management.

## Data Availability

The original contributions presented in this study are included in this article/Supplementary material, further inquiries can be directed to the corresponding authors.
