# Supplementary material for: Exercise prescription for axial spondyloarthritis: a systematic review and meta-analysis of randomized controlled trials
Source: Front Med (Lausanne). 2026 Feb 20;13:1783569. doi: 10.3389/fmed.2026.1783569 (PMC12963302; doi:10.3389/fmed.2026.1783569)

Supplementary Material

**Supplementary Figure 1.** Risk of bias assessment for each included study in the review.

**Supplementary Figure 2.** Sensitivity analysis of BASFI score.

**Supplementary Figure 3.** Sensitivity analysis of BASDAI score.

**Supplementary Figure 4.** Sensitivity analysis of BASMI score.

**Supplementary Figure 5.** Sensitivity analysis of ASDAS score.

**Supplementary Figure 6.** Sensitivity analysis of Thoracic expansion capacity.

**Supplementary Figure 7.** Sensitivity analysis of Fatigue score.

**Supplementary Figure 1.** Risk of bias assessment for each included study in the review.


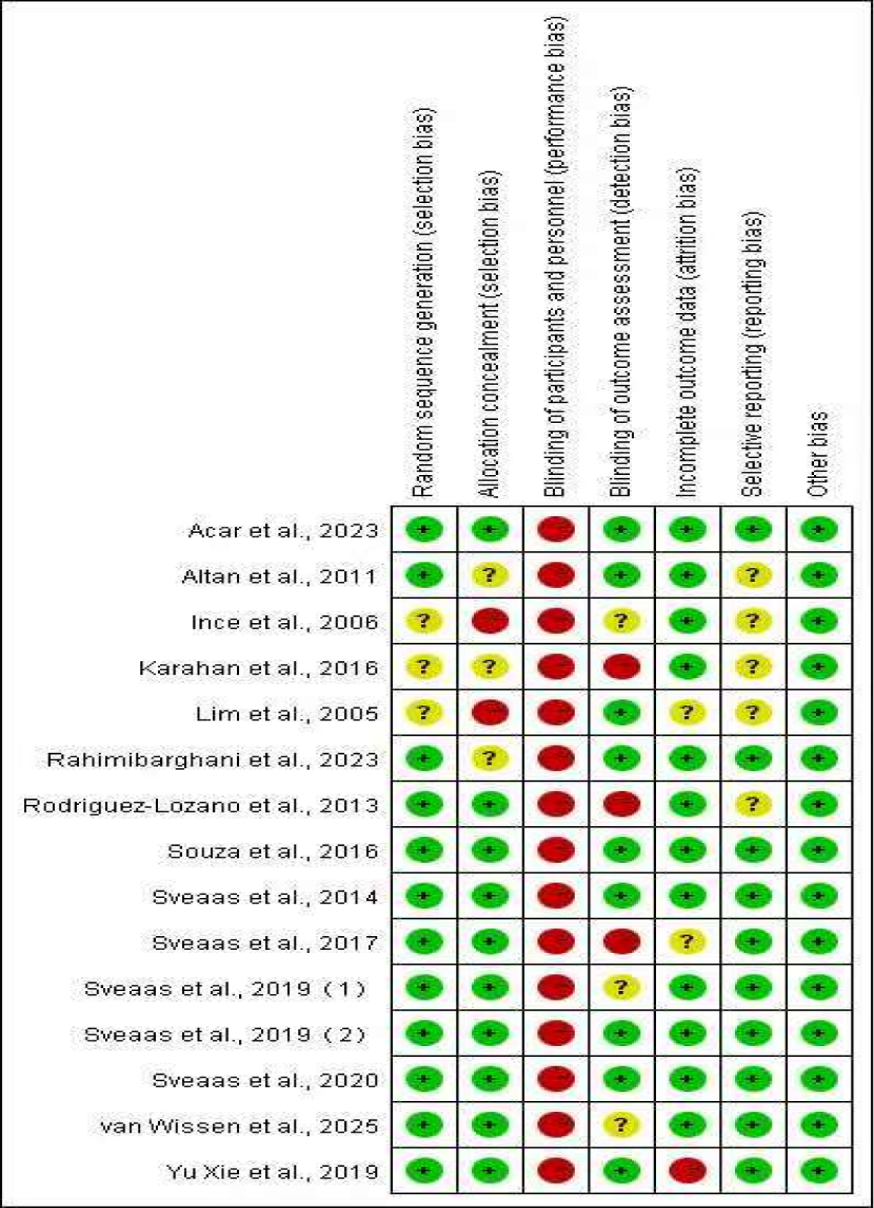


**Supplementary Figure 2.** Sensitivity analysis of BASFI score.


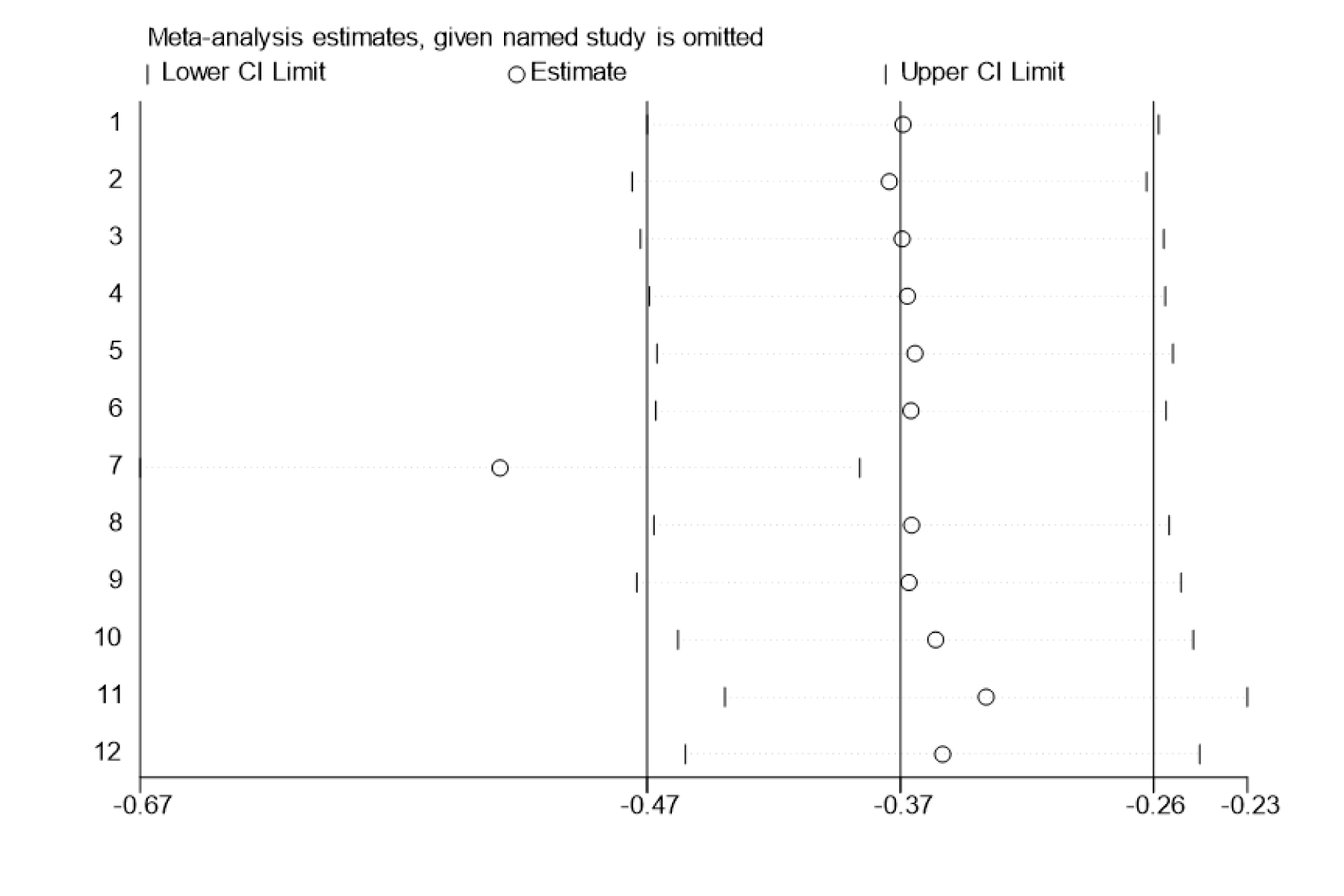


**Supplementary Figure 3.** Sensitivity analysis of BASDAI score.


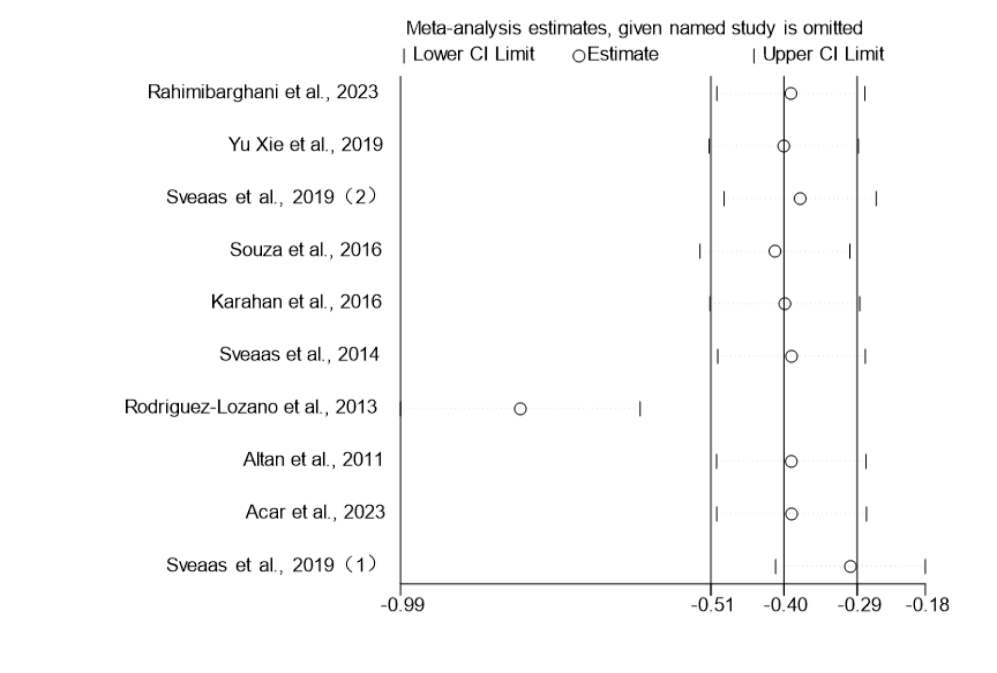


**Supplementary Figure 4.** Sensitivity analysis of BASMI score.


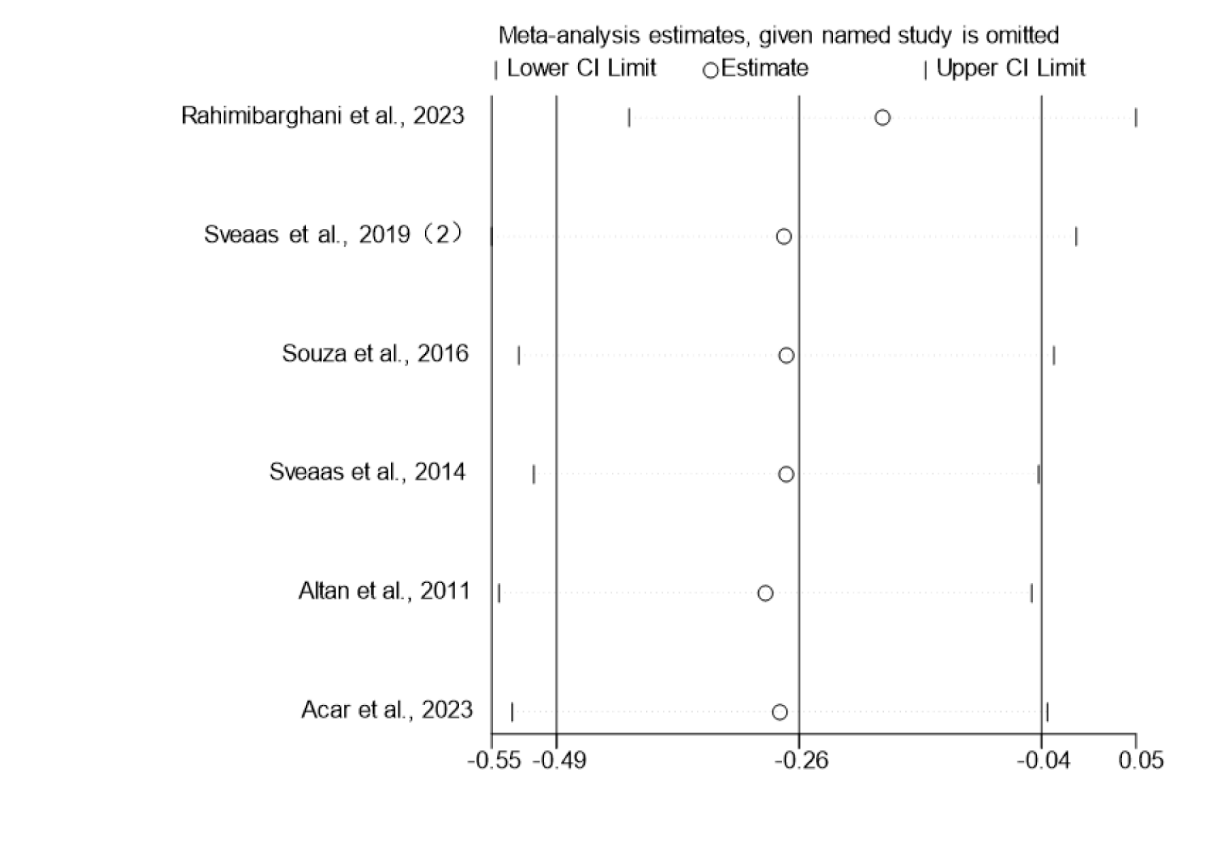


**Supplementary Figure 5.** Sensitivity analysis of ASDAS score.


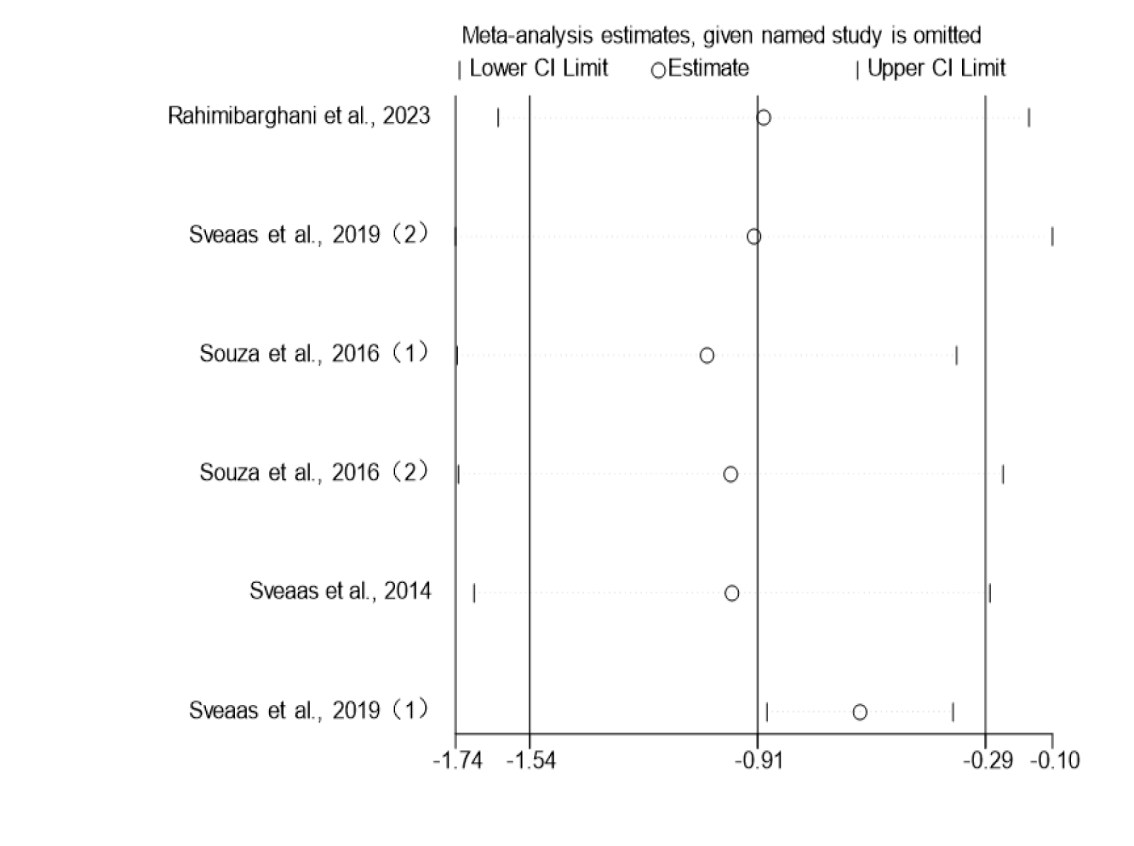


**Supplementary Figure 6.** Sensitivity analysis of Thoracic expansion capacity.


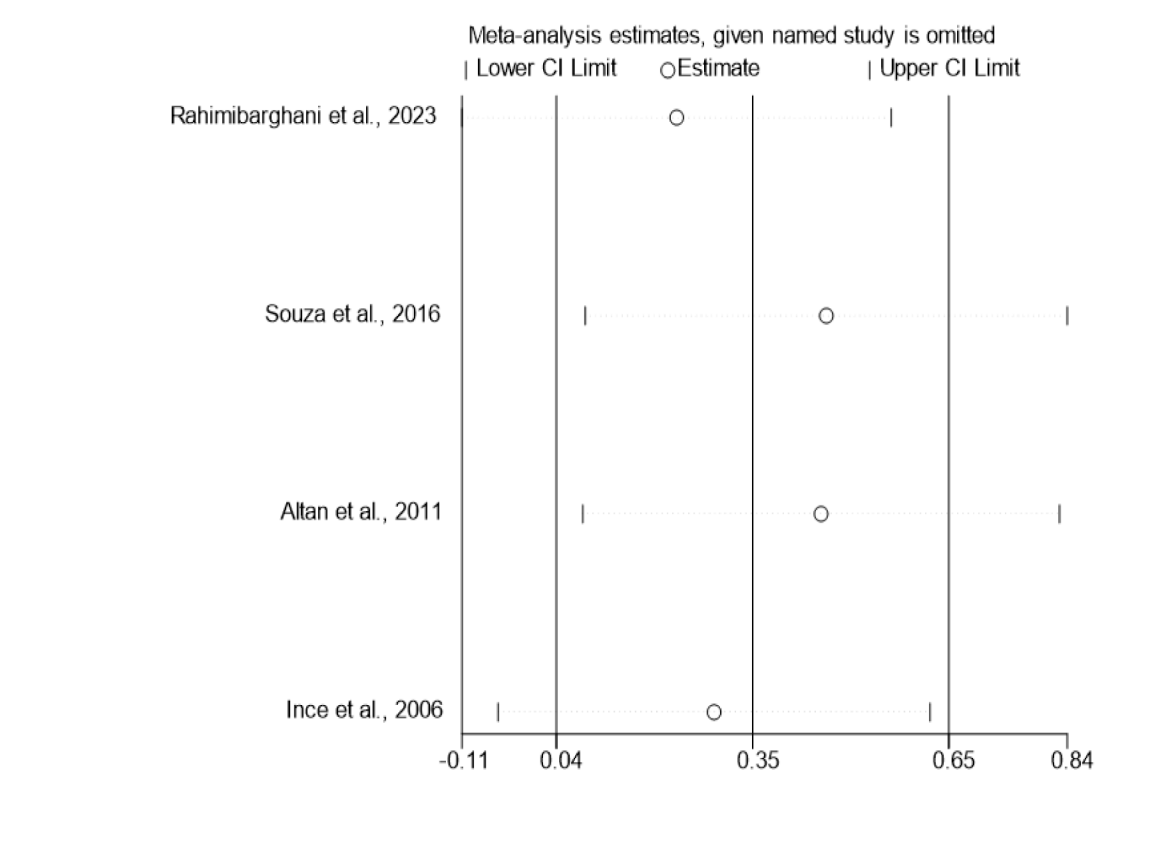


**Supplementary Figure 7.** Sensitivity analysis of Fatigue score.


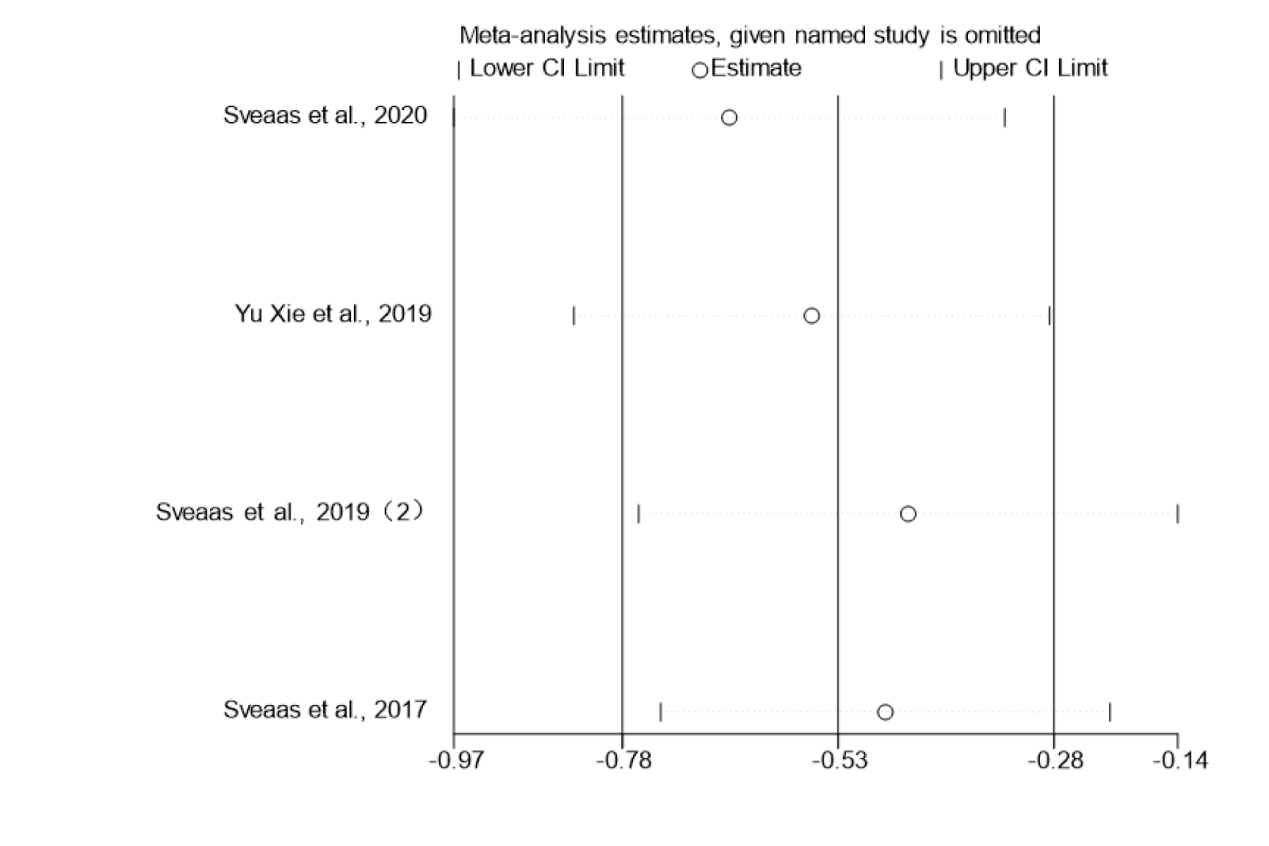

Supplement: Supplementary file 1 [file Data_Sheet_1.docx]
